# The Power of Feedback Revisited: A Meta-Analysis of Educational Feedback Research

**DOI:** 10.3389/fpsyg.2019.03087

**Published:** 2020-01-22

**Authors:** Benedikt Wisniewski, Klaus Zierer, John Hattie

**Affiliations:** ^1^Department of School Pedagogy, University of Augsburg, Augsburg, Germany; ^2^Melbourne Graduate School of Education, University of Melbourne, Parkville, VIC, Australia

**Keywords:** feedback, student learning, student achievement, meta-analysis, teaching

## Abstract

A meta-analysis (435 studies, *k* = 994, *N* > 61,000) of empirical research on the effects of feedback on student learning was conducted with the purpose of replicating and expanding the *Visible Learning* research (Hattie and Timperley, 2007; Hattie, 2009; Hattie and Zierer, 2019) from meta-synthesis. Overall results based on a random-effects model indicate a medium effect (*d* = 0.48) of feedback on student learning, but the significant heterogeneity in the data shows that feedback cannot be understood as a single consistent form of treatment. A moderator analysis revealed that the impact is substantially influenced by the information content conveyed. Furthermore, feedback has higher impact on cognitive and motor skills outcomes than on motivational and behavioral outcomes. We discuss these findings in the light of the assumptions made in *The power of feedback* (Hattie and Timperley, 2007). In general, the results suggest that feedback has rightly become a focus of teaching research and practice. However, they also point toward the necessity of interpreting different forms of feedback as independent measures.

## Introduction

Feedback is information provided by an agent regarding aspects of one’s performance or understanding (Hattie and Timperley, 2007). There is an extensive body of research on this subject: Kluger and de Nisi (1996) conducted among the most comprehensive review, based on 131 studies, over 12,000 participants, with an average effect of 0.38, noting that about a third of the effects were negative. More specifically, in the classroom domain, Hattie and Timperley (2007), Hattie (2009), and Hattie and Zierer (2019) conducted meta-syntheses relating to the effects of feedback on student achievement (which we refer to as *Visible Learning* research). These indicated a high effect (between 0.70 and 0.79) of feedback on student achievement in general. However, the authors noted the considerable variance of effects, identifying those forms of feedback as powerful that aid students in building cues and checking erroneous hypotheses and ideas, resulting in the development of more effective information processing strategies and understanding (Hattie and Timperley, 2007).

Given the impact of the *Visible Learning* research (over 25,000 citations on Google Scholar), it is important to ask whether the results presented on the effectiveness of feedback and the variables which moderate this effectiveness will stand up to scrutiny. A comprehensive meta-analysis on educational feedback which integrates the existing primary studies is still a desiderate.

### Key Proposals of the *Visible Learning* Research

Sadler (1989) claimed that the main purpose of feedback is to reduce discrepancies between current understandings and performance and a goal. From this, Hattie and Timperley (2007) argued that feedback can have different perspectives: “feed-up” (comparison of the actual status with a target status, providing information to students and teachers about the learning goals to be accomplished), “feed-back” (comparison of the actual status with a previous status, providing information to students and teachers about what they have accomplished relative to some expected standard or prior performance), and “feed-forward” (explanation of the target status based on the actual status, providing information to students and teachers that leads to an adaption of learning in the form of enhanced challenges, more self-regulation over the learning process, greater fluency and automaticity, more strategies and processes to work on the tasks, deeper understanding, and more information about what is and what is not understood). Additionally, feedback can be differentiated according to its level of cognitive complexity: It can refer to a task, a process, one’s self-regulation, or one’s self. Task level feedback means that someone receives feedback about the content, facts, or surface information (How well have the tasks been completed and understood? Is the result of a task correct or incorrect?). Feedback at the level of process means that a person receives feedback on the strategies of his or her performance. Feedback at this level is aimed at the processing of information that is necessary to understand or complete a certain task (What needs to be done to understand and master the tasks?). Feedback at the level of self-regulation means that someone receives feedback about the individual’s regulation of the strategies they are using to their performance. In contrast to process level feedback, feedback on this level does not provide information on choosing or developing strategies but to monitor the use of strategies in the learning process. It aims at a greater skill in self-evaluation or confidence to engage further on a task (What can be done to manage, guide and monitor your way of action?). The self-level focuses on the personal characteristics of the feedback recipient (often praise about the person). One of the arguments about the variability is that feedback needs to focus on the appropriate question and level of cognitive complexity, if not the message can easily be ignored, misunderstood and of low value to the recipient. Generally, it has been shown that the majority of feedback in classes is task feedback, the most received and interpreted is about “where to next,” and the least effective is self or praise feedback (Hattie and Timperley, 2007).

### Effectiveness of Feedback

Hattie and Timperley (2007) made basic assumptions with respect to variables that moderate the effectiveness of feedback on student achievement. The type of feedback was found to be decisive, with praise, punishment, rewards, and corrective feedback all having low or low to medium effects on average, but corrective feedback being highly effective for enhancing the learning of new skills and tasks. With regard to the feedback channel, video/audio and computer-assisted feedback were compared. For both forms, the synthesis showed medium high to high effects. It was also noted that specific written comments are more effective than providing grades. Hattie and Timperley (2007) also investigated the timing of feedback (immediate/delayed) and the valence (positive/negative feedback), reporting inconsistent results. It was proposed that forms of feedback with a lack of information value have low effects on student achievement.

### Methodological Considerations

As noted, the major research method in the *Visible Learning* research is synthesizing meta-analyses. The unit of analysis was the individual meta-analysis and each meta-analysis was given the same weight, regardless of the number of studies or sample size, using a fixed-effect model for the integration. This approach allows to make general assumptions about the effectiveness of feedback without the need to look at every single primary study but brings with it some restrictions addressed in the following:

Firstly, the use of a fixed-effect model may not be appropriate. A meaningful interpretation of the mean of integrated effects with this model is only possible if these effects are homogenous (Hedges and Olkin, 1985). Because previous research on feedback includes studies that differ in variants of treatment, age of participants, school type, etc., it is highly likely that the effect size varies from study to study, which is not taken into account by a fixed-effect model. By contrast, under the random-effects model, we do not assume one true effect but try to estimate the mean of a distribution of effects. The effect sizes of the studies are assumed to represent a random sample from a particular distribution of these effect sizes (Borenstein et al., 2010). The random-effects model incorporates the systematic variation of effect sizes into the weighting scheme assuming the variation to depend on factors that are unknown or that cannot be taken into account. Using the random-effects model, the variance for each primary study is in most cases larger than under the fixed-effect model because it consists of the fixed-effect variance plus a variance component τ^2^. This results also in larger confidence intervals.

Secondly, a source of distortion when using a synthesis approach results from overlapping samples of studies. By integrating a number of meta-analyses dealing with effects of feedback interventions without checking every single primary study, there is a high probability that the samples of primary studies integrated in these meta-analyses are not independent of each other, but at least some primary studies were integrated in more than one meta-analysis. Therefore, these would have to be considered as duplets–primary studies that are included in the result of the synthesis more than once–and consequently cause a distortion. In contrast to meta-synthesis, a meta-analytical approach allows to remove duplets and therefore prevent a distortion of results.

The question arises, whether synthesizing research on feedback on different levels, from different perspectives and in different directions and compressing this research in a single effect size value leads to interpretable results. In contrast to a synthesis approach, the meta-analysis of primary studies allows to weigh study effects, consider the issues of systematic variation of effect sizes, remove duplets, and search for moderator variables based on study characteristics. Therefore, a meta-analysis is likely to produce more precise results.

### Research Questions

One of the most consistent findings about the power of feedback is the remarkable variability of effects. The existing research has identified several relevant moderators like timing and specificity of the goals and task complexity (Kluger and DeNisi, 1996) and sought to understand how recipients (e.g., students, teachers) receive and understand feedback, how to frame feedback to maximize this reception, and the more critical aspects of feedback that optimize its reception and use (Hattie and Clarke, 2018; Brooks et al., 2019).

The purpose of the present study was to integrate the primary studies that provide information on feedback effects on student learning (achievement, motivation, behavior), with a meta-analytic approach that takes into account the methodological problems described in the previous part and to compare the results to the results of the *Visible Learning* research. Therefore, the study also investigates the differences between meta-synthesis and meta-analysis.

In particular, our study addressed the following research questions:

RQ1: What is the overall effect of feedback on student learning based on an integration of each of the primary studies within each of the all meta-analyses used in the *Visible Learning* research?

RQ2: To what extent is the effect of feedback moderated by specific feedback characteristics?

## Method

### General Procedure

This meta-analysis is a quantitative integration of empirical research comparing the effects of feedback on student learning outcomes. The typical strategy is (1) to compute a summary effects for all primary studies, (2) to calculate the heterogeneity of the summary effect, and (3) in case of heterogeneity between studies to identify study characteristics (i.e., moderators) that may account for a part of or all of that heterogeneity. In detail, and as suggested by Moher et al. (2009), we

• specified the study and reported characteristics making the criteria for eligibility transparent,
• described all information sources and the process for selecting studies,
• described methods of data extraction from the studies,
• described methods used for assessing risk of bias of individual studies,
• stated the principal summary measures,
• described the methods of handling data and combining results of studies, and
• described methods of additional analyses (sensitivity and moderator analyses).

**FIGURE 1:**
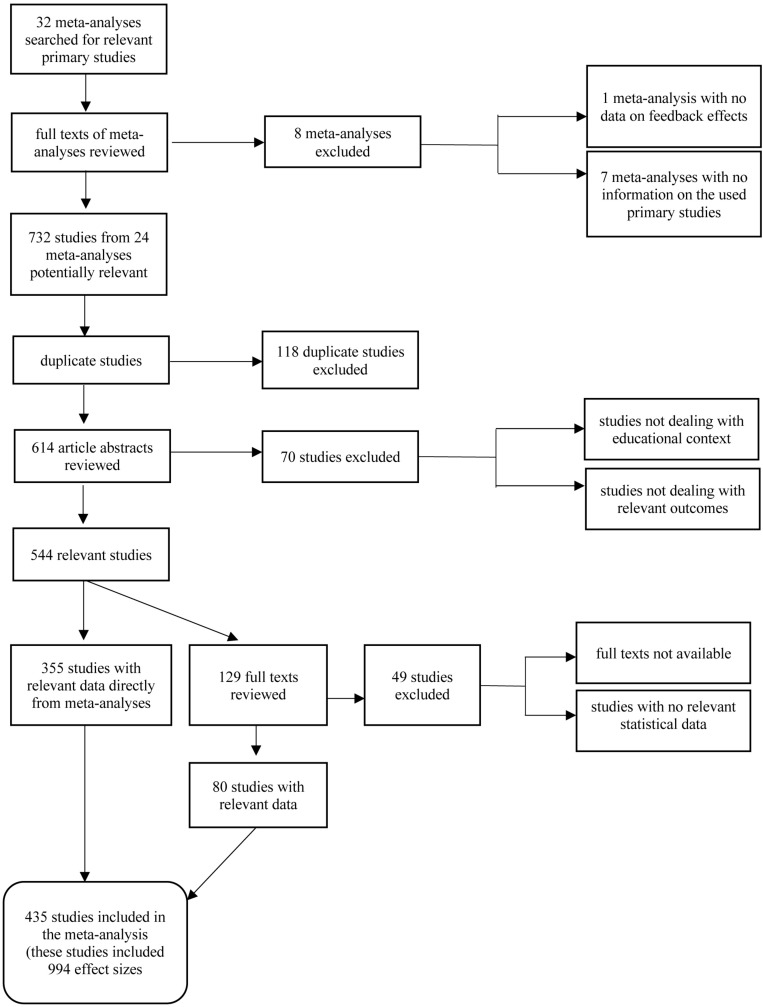
Flow diagram of study identification and selection.

The following procedure was employed in this review (see Figure 1): First, we identified primary studies from existing meta-analyses and decided whether to include these based on four inclusion criteria. Then we developed a coding scheme to compare the effects of different feedback interventions. In the next step, we defined an effect size for each primary study or study part, either by extracting it from an existing meta-analysis or (when this was not possible) calculating it from information provided in the respective primary study.

We used the random-effects model for integration of the effect sizes that met our inclusion criteria to calculate an average effect size for all studies, and, in a next step, for subgroups defined by our coding scheme. We checked for heterogeneity across the studies and conducted outlier analysis and moderator analysis to assist in reducing the heterogeneity of effect sizes.

### Identification of Studies and Inclusion Criteria

To identify the primary studies for our meta-analysis, we searched 32 existing meta-analyses that were used in the context of the *Visible Learning* research for information on primary studies that included relevant data for integration (effect sizes, sample sizes). To be included, each study had to meet the following inclusion criteria: It had to

• contain an empirical comparison of a form of feedback intervention between an experimental and a control group, or a pre-post comparison;
• report constitutive elements to calculate an effect size (e.g., include means, standard deviations, and sample sizes)
• report at least one dependent variable related to student learning (achievement, motivation, or behavioral change) and
• have an identifiable educational context (data obtained with samples of students or teachers in a kindergarten, primary school, secondary school, college or university)

The inclusion criteria are comparable to the criteria that were used to include meta-analyses in the *Visible Learning* research syntheses but allow to exclude studies from meta-analyses that encompass both an educational and a non-educational context (Wiersma, 1992; Kluger and DeNisi, 1996; Standley, 1996).

We included studies with controlled designs as well as pre-post-test designs, and this became a moderator to investigate any differences related to design (Slavin, 2008). Whenever existing meta-analyses reported the relevant statistical data from the primary studies, we used this data. When no relevant statistical data from primary studies were reported, we contacted the authors of the meta-analyses via e-mail and asked to provide the missing information. Four authors responded and three of them provided the effect sizes and sample sizes of the primary studies which they used in their meta-analysis. When no relevant data were reported in a meta-analysis and authors didn’t provide them, we reconstructed the effect sizes directly from the primary studies whenever possible. Some studies were excluded, either because we could not reconstruct their effect size, or because of other reasons as specified in Table 1.

**TABLE 1:** Existing meta-analyses investigating the factor feedback.

**Authors**	**Year**	**Status**	**Access to effect sizes and sample sizes**
Azevedo and Bernard	1995	Included	From meta-analysis
Bangert-Drowns et al.	1991	Included	From meta-analysis
Biber et al.	2011	Included	From meta-analysis
Brown	2014	Excluded	Data not available
Getsie et al.	1985	Excluded	No effect sizes indicated; no individual references provided
Graham et al.	2015	Partially included	From meta-analysis
Kang and Han	2015	Included	From meta-analysis
Kluger and DeNisi	1996	Partially included	Original data received from authors; studies that do not deal with educational context excluded
Kulik and Kulik	1988	Partially included	33 sample size values missing; reconstruction from the primary studies not possible
L’Hommedieu et al.	1990	Included	From meta-analysis
Li	2010	Included	From meta-analysis
Lysakowski and Walberg	1980	Excluded	No effect sizes indicated, effect sizes and sample sizes not reconstructable from original studies
Lysakowski and Walberg	1981	Partially included	44 of 54 primary studies excluded because they do not deal with feedback on the relevant outcomes effect sizes reconstructed from primary studies
Lyster and Saito	2010	Partially included	Effect sizes reconstructed from original studies
Menges and Brinko	1986	Partially included	Missing values reconstructed from primary studies
Miller	2003	Included	Updated set of studies was used (Miller and Pan, 2012), instead of the eleven effects from eight studies used by Miller (2003), 31 effects from 13 studies were integrated
Neubert	1998	Partially included	Effect sizes/sample sizes reconstructed from primary studies
Rummel and Feinberg	1988	Partially included	38 of 45 studies excluded because they do not deal with the relevant outcomes
Russell and Spada	2006	included	From meta-analysis
Schimmel	1983	Excluded	Data not available
Skiba, Casey, and Center	1985	Excluded	Data no longer available (even directly from authors)
Standley	1996	Partially included	82 of 98 studies excluded because they do not deal with a school context
Swanson and Lussier	2001	Partially included	Effect sizes/sample sizes reconstructed from primary studies
Tenenbaum and Goldring	1989	Included	Statistical data from meta-analysis, but no references of integrated studies provided
Travlos and Pratt	1995	Partially included	From meta-analysis
Truscott	2007	Included	From meta-analysis
van der Kleij et al.	2015	Included	From meta-analysis
Walberg	1982	Excluded	No effect sizes and sample sizes indicated; reconstruction of data no longer possible
Wiersma	1992	Partially included	10 of 20 studies excluded because they do not deal with an educational context
Wilkinson	1981	Excluded	Data not available
Witt et al.	2006	Excluded	No data on feedback effects
Yeany and Miller	1983	Partially included	45 of 49 studies excluded because data was not reconstructable

### Coding of Study Features

To be able to identify characteristics that influence the impact of feedback, a coding scheme was developed. It includes the following categories of study features: publication type (i.e., journal article, dissertation), outcome measure (i.e., cognitive, motivational, physical, behavioral), type of feedback (i.e., reinforcement/punishment, corrective, high-information), feedback channel (i.e., written, oral, video-, audio- or computer-assisted), and direction (i.e., teacher > learner, learner > teacher). Some of the study features of interest had to be dropped (i.e., perspective of feedback, way of measuring the outcome) because there were insufficient data, or the feature could not be defined based on the article abstracts. Generally, the study features for our coding scheme are orientated toward Hattie’s and Timperley’s (2007) coding features.

We analyzed inter-coder consistency to ensure reliability among coders by randomly selecting 10% of the studies and having them coded separately by two coders. Based on this, we assessed intercoder reliability of each coding variable. For the 6 moderator variables, Krippendorff’s alpha ranged from 0.81 to 0.99, and therefore above the acceptable level (Krippendorff, 2004). The two coders then discussed and resolved remaining disagreements and established an operational rule that provided precise criteria for the coding of studies according to each moderator variable. The lead author then used these operational rules to code the rest of the studies.

### Calculation of Effect Sizes

For the computation of effect sizes, tests for heterogeneity, and in the analysis of moderator variables, we used the Meta and Metafor packages for R (R Core Team, 2017). To compare study results, Cohen’s (1988) *d* effect size measure was applied. This is calculated as

(1) $$d=\frac{{\bar{X}}_{1}-\,{\bar{X}}_{2}}{\sigma_{p\,o\,o\,l}}$$

with the pooled standard deviation of

(2) $$\sigma \,s_{p\,o\,o\,l}=\sqrt{\frac{\left(n_{1}-1\right)\,\sigma_{1}^{2}+\left(n_{2}-1\right)\,\sigma_{2}^{2}}{n\,1+n\,2-2}}$$

Hedges and Olkin (1985) demonstrated that the unsystematic error variance of a primary study is determined by the variance of the effect size. The higher the variance, the less precise the study effect. Because study effects that have higher precision are to be weighted more strongly than effects that have lower precision, the inverse of the variance of the study effect in relation to the inverse of the sum of the variance inverse values of all k primary studies serves as a correction factor (Rustenbach, 2003). The inverse variance weight is calculated as

(3) wi=⁢1σd⁢i    2∑i=1k1σd⁢i    2

The average weighted effect size *d* is the sum of all weighted effect sizes of the k primary studies. In the fixed-effect model, the variance $\sigma_{{\text{d}}_{∙}}^{2}$ equals $\nu_{i}^{2}$, which is derived from the individual study variances (5). In the random-effects model, the variance $\sigma_{{\text{d}}_{∙}}^{2}$ consists of a first component $\nu_{i}^{2}$ (5) and a second component, τ^2^ (6), which is the variance of the effect size distribution.

(4) $$\sigma_{{\text{d}}_{∙}}^{2}=\,\nu_{i}^{2}+\,\tau^{2}$$

(5) νi2=⁢1∑i=1k1σd⁢i    2

(6) $$\tau^{2}=\,\frac{Q-\left(k-1\right)\,}{\sum w_{i}-\frac{\sum w_{i}^{2}}{\sum w_{i}}}$$

### Integration Model

The model of random effects (Hedges and Vevea, 1998) was used to integrate the study effects. With a random-effects model we attempted to generalize findings beyond the included studies by assuming that the selected studies are random samples from a larger population (Cheung et al., 2012). Consequently, study effects may vary within a single study and between individual studies, hence no common population value is assumed.

The random-effects model takes two variance components into account. These are the sum of the individual standard errors of the study effects resulting from the sample basis of the individual studies, and the variation in the random selection of the effect sizes for the meta-analysis. A meaningful interpretation of average effect sizes from several primary studies does not necessarily require homogeneity (i.e., that the variation of the study effects is solely random, Rustenbach. 2003). The basic assumption here is that differences in effect sizes within the sample are due to sample errors as well as systematic variation.

The integration of multiple effect sizes does not only require independence of the primary studies included in the meta-analyses, but also independence of the observed effects reported in the primary studies. The second assumption is violated when sampling errors and/or true effects are correlated. This can be the case when studies report more than one effect and these effects stem from comparisons with a common control group (multiple treatment studies, Gleser and Olkin, 2009). To adequately integrate statistically dependent effect sizes, there are different approaches, for example selecting one effect size per study, averaging all effects reported in one study, or conducting multivariate meta-analysis (which requires knowledge of the underlying covariance structure among effect sizes). If a study reported more than one effect size and the multiple outcomes could not be treated as independent from each other (because they used one common sample), we accounted for this non-independence by robust variance estimation (RVE, Sidik and Jonkman, 2006; Hedges et al., 2010). This method allows the integration of statistically dependent effect sizes within a meta-analysis without knowledge of the underlying covariance structure among effect sizes.

### Bias and Heterogeneity

Possible selection bias was tested by the means of a funnel plot, a scatter diagram that plots the treatment effect on the *x*-axis against the study size on the y-axis, and the means of a normal-quantile-plot, in which the observed effect sizes are compared with the expected values of the effect sizes drawn from a normal distribution. Additionally, Egger’s et al. (1997) regression test was used to detect funnel plot asymmetry.

A *Q*-test (Shadish and Haddock, 1994) was performed to test the homogeneity of the observed effect sizes.

(7) $$\text{Q}=\,\sum_{i=1}^{k}\frac{{\left(d_{i}-d_{∙}\right)}^{2}}{\sigma_{d\,i}^{2}}\,$$

The test variable Q is χ^2^-distributed with degrees of freedom of the number k-1. Q can be used to check whether effect sizes of a group are homogeneous or whether at least one of the effect sizes differs significantly from the others. In order to be able to provide information about the degree of heterogeneity, *I*^2^was computed (Deeks et al., 2008). *I*^2^ is a measure of the degree of heterogeneity among a set of studies along a 0% – 100% scale and can be interpreted as moderate for values between 30 and 60%, substantial for values over 50%, and considerable for values over 75% (Deeks et al., 2008).

### Outlier Analysis

By definition, no outliers exist in the random-effects model because the individual study effects are not based on a constant population mean. Extreme values are attributed to natural variation. An outlier analysis, however, can serve to identify unusual primary studies. We used the method of adjusted standardized residuals to determine whether effect sizes have inflated variance. An adjusted residual is the deviation of an individual study effect from the adjusted mean effect, i.e., the mean effect of all other study effects. Adjusted standardized residuals follow the normal distribution and are therefore significantly different from 0 when they are >1.96. They are conventionally classified as extreme values when > 2 (Hedges and Olkin, 1985).

### Moderator Analysis

For heterogeneous data sets, suitable moderator variables must be used for a more meaningful interpretation. In extreme cases, this can lead to a division into k factor levels if none of the primary studies can be integrated into a homogeneous group. Q_*B*_ reflects the amount of heterogeneity that can be attributed to the moderator variable, whereas Q_*W*_ provides information on the amount of heterogeneity that remains within the moderator category. The actual suitability of a moderator variable within a fixed-effect model is demonstrated by the fact that homogeneous effect sizes are present within the primary study group defined by it (Q_W*empirical*_ < Q_W*critical*_) and at the same time the average effect sizes of the individual groups differ significantly from each other (Q_B*empirical*_ > Q_B*critical*_). If both conditions are fulfilled, homogeneous factor levels are present, which are defined by moderator variables, leading to a meaningful separation of the primary studies. However, by this definition, homogeneity of effect sizes within hypothesized moderator groups will occur rarely in real data, which means that fixed-effect models are rarely appropriate for real data in meta-analyses and random-effects models should be preferred (Hunter and Schmidt, 2000). In random-effects models, it can be tested if moderators are suitable for reducing heterogeneity (the random-effects model then becoming a mixed-effects model), but without assuming homogeneity (Viechtbauer, 2007). Therefore, we used the article abstracts of the primary studies to define meaningful moderator variables and set the moderator values for each primary study according to our coding scheme. The following moderators were used:

#### Research Design

Studies with control groups were separated from studies with a pre-post-test design. Effect sizes from pre-post designs are generally less reliable and less informative about the effects of the intervention because they are likely to be influenced by confounding variables (Morris and DeShon, 2002).

#### Publication Type

The type of publication (journal article or dissertation) was used as a moderator. Published studies may be prone to having larger effect sizes than unpublished studies because they are less likely to be rejected when they present significant results (Light and Pillemer, 1986).

#### Outcome Measure

The *Visible Learning* research investigated the impact of the factor feedback on student achievement. However, not all primary studies that were integrated in the meta-analyses contain an achievement outcome measure. Consequently, for our meta-analysis, we differentiated between four types of outcome measure: cognitive (including student achievement, retention, cognitive test performance), motivational (including intrinsic motivation, locus of control, self-efficacy and persistence), physical (development of motor skills) and behavioral (student behavior in classrooms, discipline).

#### Type of Feedback

A further distinction was made between different types of feedback, namely reinforcement/punishment, corrective feedback, and high-information feedback. Forms of reinforcement and punishment apply pleasant or aversive consequences to increase or decrease the frequency of a desired response or behavior. These forms of feedback provide a minimum amount of information on task level and no information on the levels of process or self-regulation. Corrective forms of feedback typically contain information about the task level in the form of “right or wrong” and the provision of the correct answer to the task. Feedback not only refers to how successfully a skill was performed (knowledge on result), but also to how a skill is performed (knowledge of performance). For some forms of feedback, i.e., modeling, additional information is provided on how the skill could be performed more successfully. Feedback was classified as high-information feedback when it was constituted by information as described for corrective feedback and additionally contained information on self-regulation from monitoring attention, emotions, or motivation during the learning process.

#### Feedback Channel

Some studies investigated the effects of feedback according to the channel by which it is provided. Hence, the distinction between three forms: oral, written, and video-, audio- or computer-assisted feedback.

#### Feedback Direction

This moderator refers to who gives and who receives feedback. We differentiated between feedback that is given by teachers to students, feedback that is given by students to teachers, and feedback that is given by students to students.

## Results

### Identification of Studies

Our search strategy yielded 732 primary studies (see Figure 1). After the selection process, in the final data set, 994 effect sizes from 435 studies (listed in the Supplementary Appendix), including about 61,000 subjects, were used for our meta-analysis.

**FIGURE 2:**
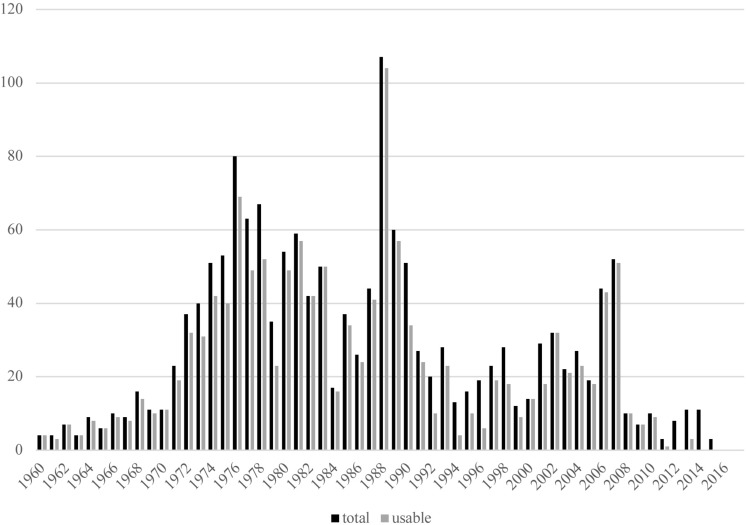
Number of study effects per year.

Figure 2 shows the distribution of the included effect sizes related to the years of publication. The median of publication year is 1985. Fifteen percent of the integrated effect sizes are taken from studies published in the last 15 years.

### General Impact of Feedback

The integration of all study effects with the random-effects model leads to a weighted average effect size of *d* = 0.55. 17% of the effects were negative. The confidence interval ranges from 0.48 to 0.62. Cohen’s *U*3, the percentage of those scores in the experimental groups that exceed the average score in the control groups is 70%. The probability of homogenous effects is <0.001 with *Q* = 7,339 (*df* = 993) and *I*^2^ = 86.47%.

### Bias and Heterogeneity

**FIGURE 3:**
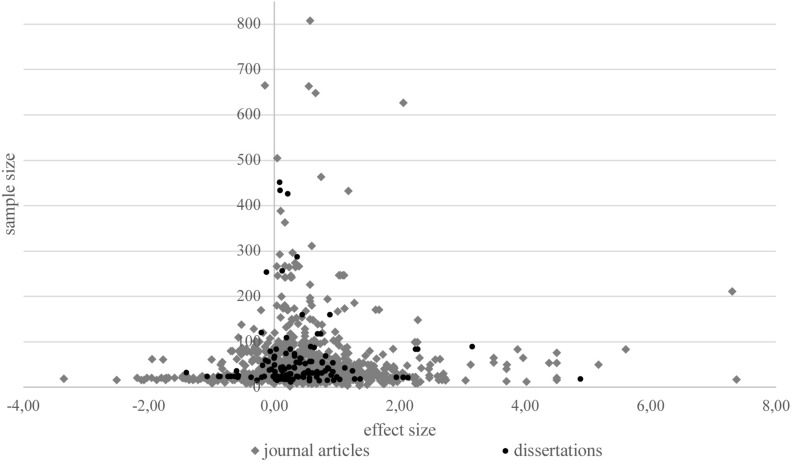
Funnel plot of all study effects.

**FIGURE 4:**
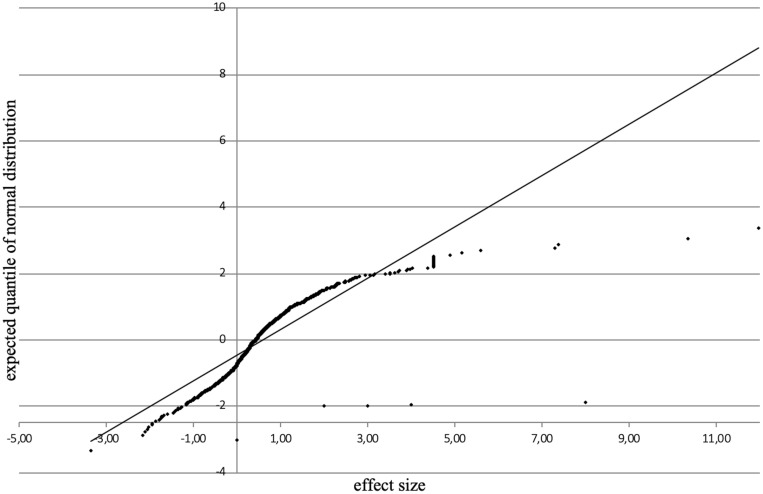
Normal-quantile-plot of all study effects.

In the funnel plot (Figure 3) all feedback effects are plotted on the x-axis against the study sizes on the *y*-axis. The funnel plot is a visual aid to identify conspicuous data characteristics, producing a symmetric inverted funnel for data in which bias and systematic heterogeneity are unlikely. The normal-quantile-plot (Figure 4) compares the effect sizes from the primary studies with hypothetical values predicted for a standard normal distribution. It indicates that the existing data shows unexpected distribution characteristics. The funnel plot displays an asymmetric distribution of effect sizes for the whole sample and there is an unusually large range of effect sizes and an unusually large number of extreme effect size values. This is confirmed by Egger’s et al. (1997) test for funnel plot asymmetry (*z* = 9.52, *p* < 0.001). However, the asymmetry is only produced by the effect sizes from studies published in journals (*z* = 9.75, *p* < 0.0001), while there is no asymmetry for effect sizes from dissertation articles (*z* = 1.03, *p* = 0.30). The normal-quantile-plot hints at more extreme values than would be expected from a normal distribution of effect sizes. The plot is markedly non-linear (i.e., the points do not approximately lie on the regression line), making it implausible that the data come from a normal distribution.

### Outlier Analysis

Thirty-five (3.5%) of all effect sizes were identified as extreme values (standardized residuals > 2) and excluded. An exclusion of these extreme values leads to a reduction of the average weighted effect size to 0.48 (CL*:* 0.44–0.51, *Q* = 5,771.43, *df* = 958, *I*^2^ = 83.40%).

The most extreme values were found in the meta-analysis by Standley (1996). This meta-analysis deals with a special form of feedback, namely music as reinforcement for education/therapy objectives. The author reports five effect sizes larger than 5, two of them larger than 10 for the educational context (the effect sizes for the therapeutical context were not considered in our meta-analysis). These effect sizes must be classified as gigantic. In an educational context, effect sizes that large are uncommon and much higher than usually expected from any treatment. Following Brand and Bradley (2012) they can be called “voodoo” effects. For example. for one primary study with gigantic effects (Madsen et al., 1976), Standley calculated four effect sizes of 1.74, 3.88, 5.60, and 11.98. Madsen et al. (1976) report a pre-post-test ANOVA with four groups (*F* = 7.54, *df* = 3.76, *p* < 0.1), a Newman–Keuls multiple range comparison of mean pre-posttest differences scores (5.2; 3.5; 0.95; 0.90) and the means of two experimental and two control groups (*n* = 20 in all groups) regarding their results in a math test. Because Madsen et al. (1976) do not report standard deviations, it is unclear how the respective effect sizes were calculated by Standley (1996) and a re-calculation was impossible. Most importantly, though, it remains unclear why four effect sizes are reported, given the fact that the primary study uses two control and two experimental groups. Another study for which Standley’s meta-analysis finds gigantic effect sizes is Madsen et al. (1975), which reports a pre-pos-test ANOVA (*F* = 3.18, *df* = 2, *p* > 0.05) and a pre-post × group ANOVA (*F* = 8.11, *df* = 2.72, *p* < 0.1) for two dependent variables (behavior and math test score). Again, no standard deviations are reported in the primary study and it is unclear how the effect sizes of 10.34, 3.71, 5.17, and 3.13 were calculated by Standley (1996). The author was contacted but did not respond.

### Comparison With Existing Meta-Analyses

**FIGURE 5:**
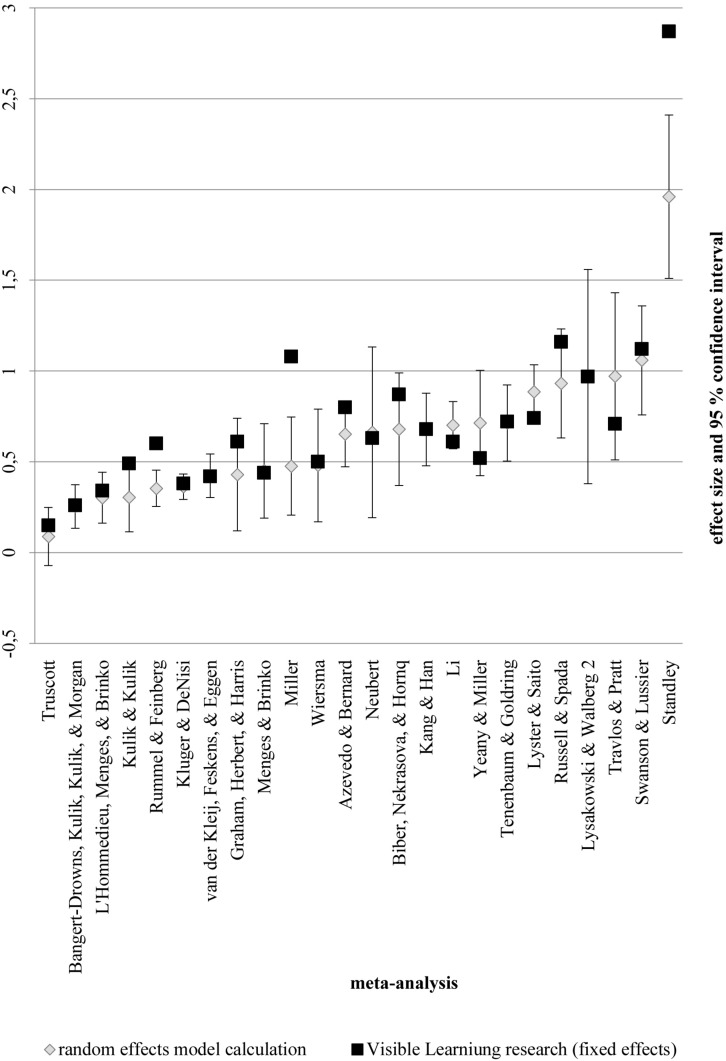
Random-effects model calculation for the subsets of previous meta-analyses.

The average effect sizes of the subsets of primary studies as used in the existing meta-analyses are shown in Figure 5. For three meta-analyses (Rummel and Feinberg, 1988; Standley, 1996; Miller, 2003), the average effect size used in the *Visible Learning* research is outside the confidence interval of our meta-analysis.

### Moderator Analysis

Table 2 shows the results of the tests for heterogeneity between and within the subgroups defined by our six moderator variables. Table 3 shows the outputs from the mixed-effects moderator analysis. Five of six moderators (research design, publication type, outcome measure, type of feedback, and feedback direction) proved to be statistically significant, the feedback channel proved to be a non-significant moderator.

**TABLE 2:** Tests of heterogeneity between and within the moderator subgroups.

**Moderator**	**Q_B_ (*df*)**	***p***	**Q_W_ (*df*)**	***p***	***I*^2^**
Research design	29.06 (1)	<0.0001	5639.74 (955)	<0.0001	83.4%
Publication type	6.15 (1)	<0.05	5699.39 (957)	<0.0001	83.4%
Outcome measure	14.12 (3)	<0.001	4380.69 (750)	<0.0001	83.0%
Feedback type	41.52 (2)	<0.0001	1541.06 (316)	<0.0001	80.9%
Feedback channel	5.12 (2)	>0.05	2218.20 (337)	<0.0001	85.2%
Feedback direction	9.35 (2)	<0.001	4695.60 (852)	<0.0001	81.9%

*Q_*B*_, heterogeneity between groups; Q_*W*_, heterogeneity within groups; *I*^2^, total amount of heterogeneity.*

**TABLE 3:** Effect sizes and heterogeneity for different moderator subgroups.

**Moderator**	***k***	***d***	**C.I.**	**Q**	***I*^2^**
**Research design**					
Controlled study	713	0.42	[0.37 – 0.46]	3321.86	78.6%
Pre-post study	244	0.63	[0.56 – 0.69]	2317.88	89.5%
**Publication type**					
Journal article	843	0.49	[0.45 – 0.53]	5176.67	83.7%
Dissertation	116	0.36	[0.25 – 0.46]	522.72	78.0%
**Outcome measure**					
Cognitive	597	0.51	[0.46 – 0.55]	3689.88	83.8%
Motivational	109	0.33	[0.23 – 0.42]	600.96	82.0%
Physical	19	0.63	[0.34– 0.92]	36.65	50.9%
Behavioral	30	0.48	[−0.09 – 1.06]	0.28	50.0%
**Type of feedback**					
Reinforcement or punishment	39	0.24	[0.06 – 0.43]	123.54	69.2%
Corrective feedback	238	0.46	[0.39 – 0.55]	1260.41	81.2%
High-information feedback	42	0.99	[0.82 – 1.15]	157.12	73.9%
**Feedback direction**					
Teacher > student	812	0.47	[0.43 – 0.51]	4510.40	82.0%
Student > teacher	27	0.35	[0.13 – 0.56]	52.92	50.9%
Student > student	16	0.85	[0.59 – 1.11]	132.28	88.7%

**k*, number of study effects; *d*, average effect size; C.I., 95% confidence interval; Q, heterogeneity within subgroup; *I*^2^, total amount of heterogeneity within subgroup.*

## Discussion

The aim of the present study was to investigate the effectiveness of feedback in the educational context with a meta-analytic approach. With *d* = 0.48 (cleared of extreme values), the overall effect of different forms of feedback on student learning is medium-high (RQ1), although the variability of the effects is most notable.

The average weighted effect size differs considerably from the results of meta-synthesis (*d* = 0.79, Hattie and Timperley, 2007). This is likely due to several factors which have been pointed out by Wecker et al. (2017): One reason for this difference could be that we excluded duplets and therefore avoided studies being double-counted. A second reason for the difference between synthesis and meta-analysis could be our weighting of the effect sizes by precision which was not applied in the *Visible Learning* research. We used a random-effects model and gave every single effect size a weight based on its variance, while the meta*-*synthesis approach is based on a fixed-effect model and sums up average effect sizes. Comparing the average effect sizes that were synthesized with the calculation conducted in this meta-analysis for the subsets of meta-analyses (Figure 5), there is basic agreement between the synthesis approach and the meta-analysis with 21 of 24 confidence intervals overlapping. but it also becomes apparent that the synthesized average mean effect contains three overestimated values. In the random-effects model, large studies lose influence and small studies gain influence (Borenstein et al., 2009). Consequently, if there is a large number of studies with large samples and high effects, the average effect size will be higher under the fixed-effect model than under the random-effects model and vice versa. As our data includes a relatively large number of small studies with high effects, the use of the random-effects model leads to a higher average effect size than the fixed-effect model (the mean would be 0.41 with a confidence interval of 0.40 – 0.43). Consequently, the difference in effect size between synthesis and meta-analysis cannot be explained by the use of a random-effects model.

We assume that the different results mainly stem from the fact that a number of studies used in the synthesis were excluded from our meta-analysis, either due to a lack of detailed information on the statistical data or due to content-related considerations (studies that did not explicitly deal with an educational context or did not report information on learning outcomes). The average effect size from this meta-analysis is based on a smaller sample of studies than the synthesis, but at the same time, the selection of studies produces more accurate results because it could be checked for each single study if it actually fulfills the inclusion criteria.

Care is needed, however, with focusing too much on the average effect size as the integrated studies show high heterogeneity, suggesting that, conform to expectations, not all feedback is the same and one (average) effect size does not fit all. The funnel- and normal-quantile-plots illustrate that the observed data does not capture the construct of feedback in an unbiased way and that there is an distribution of effect sizes which is not conform to a symmetric inverted funnel theoretically expected for data in which bias and systematic heterogeneity are unlikely. These issues and the results of the tests for homogeneity speak largely to the variability in effects and the need to search for meaningful moderators.

### Effects of Different Forms of Feedback

Heterogeneity likely results from different forms of feedback, ranging from the simplest forms of operant conditioning to elaborate forms of error modeling, from feedback to kindergarten children to feedback to university professors, from feedback that people get while learning a handstand to feedback that people get while learning a foreign language.

This study investigated six moderators (RQ2) – research design, publication type, outcome measure, type of feedback, feedback channel, and feedback direction. Generally, and conform to expectations (Light and Pillemer, 1986; Morris and DeShon, 2002), the average effect of feedback as reported in pre-post design studies is higher than in controlled studies and higher reported in published journal articles than in dissertations. Feedback effects seem to be less likely to be published when they are low or even negative.

Feedback is more effective for cognitive and physical outcome measures than for motivational and behavioral criteria. These claims must be partly interpreted with some caution because there are few studies available related to physical and behavioral outcomes, substantially reducing the precision of the average effect size. From a cognitive perspective, feedback is often considered a source of information that is necessary to improve on a task. Previous meta-analyses have produced inconsistent results of feedback on cognitive variables (Kulik and Kulik, 1988; Bangert-Drowns et al., 1991; Azevedo and Bernard, 1995; Kluger and DeNisi, 1996; Lyster and Saito, 2010) and significant heterogeneity remains within the sub-group defined by this moderator in our analysis. From a motivational perspective, feedback is mainly considered to influence dependent variables like intrinsic motivation, locus of control, self-efficacy, or persistence. For these outcomes, the average effect is low. A possible explanation from motivation theory is that feedback can have negative effects on motivation by reducing the experience of autonomy and self-efficacy when it is controlling, negative and uninformative (Ryan and Deci, 2000). Twenty one percent of the effect sizes related to motivational outcomes in our data were negative, with 86% of the feedback interventions leading to these negative effects being uninformative (rewards or punishments). Hattie and Timperley (2007) have stated that rewards significantly undermine intrinsic motivation and feedback administered in a controlling way caused negative effects, taking away responsibility from learners for motivating or regulating themselves. The results do not indicate that feedback effects on motivation *per se* are low but that effects of uninformative forms of feedback on motivation are low or even negative.

Feedback is more effective the more information it contains. Simple forms of reinforcement and punishment have low effects, while high-information feedback is most effective. Hattie and Timperley (2007) have noted the importance of not just the information in the feedback, but the appropriateness of the timing of the feedback relating to where the students are in the instructional cycle, moving from focusing on the task, the strategies underlying the task, and the self-regulation of the processes. Claims by Lysakowski and Walberg (1981) that the effects of rewards or positive reinforcement on classroom learning are strong with an average effect size of 1.17 have to be placed in the context, therefore, of being effective more at the task level (which rightly and more likely wrongly is the focus of most teacher feedback; Hattie, 2009). Miller (2003), Russel and Spada (2006), Truscott (2007), Li (2010), Kang and Han (2015) and Brown (2016) have all similarly noted that the effectiveness of corrective feedback is influenced by additional moderating variables, such as learners’ proficiency, the setting, and the genre of the task (Kang and Han, 2015). These variables were not taken into account in our meta-analysis. High-information feedback contains information on task, process and (sometimes) self-regulation level. Its effect is very large, which suggests that students highly benefit from feedback when it helps them not only to understand what mistakes they made, but also why they made these mistakes and what they can do to avoid them the next time. These results are in line with claims of Hattie and Timperley (2007) who assume forms of feedback “most useful when they assist students in rejecting erroneous hypotheses and provide direction for searching and strategizing” (pp. 91–92).

Findings by Biber et al. (2011) that written feedback is more effective than oral feedback could not be confirmed. Although there is a tendency of our results pointing in a similar direction, the feedback channel proved to be a non-significant moderator.

Only a very small percentage of the primary studies investigated feedback from students to teachers and out of these, 26 effect sizes could be used to compute an average effect size. These effects were located mainly in studies dealing with higher education, i.e., with feedback from university or college students to their professors. Consequently, the data does not allow conclusions on the effectiveness of student > teacher feedback in the K-12 context. In general, feedback from teachers to students is more effective than from students to teachers, but the average effect of student > teacher feedback has a high variance and there is a rich literature related to this variance (Marsh, 1987; Uttl et al., 2017). With respect to the direction of feedback, student-student-feedback is the most effective form, although, again care is needed as these estimates are based on a very small sample of only eight studies.

### General Limitations

We tried to shed more light on the role and variability of feedback in the educational context with the help of meta-analysis in comparison to meta-synthesis. Both approaches are often confronted with the accusation of comparing apples and oranges. Still, it is legitimate to aggregate heterogenous data in order to make general statements, but it has to be kept in mind that these statements are often the first step to later understanding the critical moderators. The *Visible Learning* research aimed to develop, present and defend a set of propositions and a story about not only the mean effects of many influences on student achievement but the variability of these means. As Hattie and Clarke (2018) have recently stated, a danger lies in over-simplifications, simply using average effect sizes, and ignoring the variability across many studies, influences, contexts, and moderators.

In this study, we used a random-effects/mixed-effects model to deal with heterogeneity of effect sizes and accounted for non-independence of study effects by RVE. Raudenbush and Bryk (1985) have recommended that the use of hierarchical linear modeling may be more optimal to account for the nesting of studies within a meta-analysis and this would improve the fidelity of the estimation.

## Conclusion

Notwithstanding, there has been a long search for the optimal measures of central tendency – and we have added another approach to better understand the power of feedback.

Feedback must be recognized as a complex and differentiated construct that includes many different forms with, at times, quite different effects on student learning. Most importantly, feedback is more effective, the more information it contains and research on estimating this information would be a valuable addition to the area. Developing models, such as the Hattie and Timperley (2007) model, would also advance the research, as such models provide a more nuanced view of feedback, aims to include the moderators, and can be refuted.

Estimates of the effects of feedback range between 0.48 (this meta-analysis), 0.70 (Hattie, 2009), and 0.79 (Hattie and Timperley, 2007) but the pursuit of seeking the optimal moderators is the core business of future research. Feedback, on average, is powerful, but some feedback is more powerful.

## Author Contributions

BW developed the idea for this manuscript and the methodological framework and performed the analytic calculations. KZ and JH verified the analytical methods and supervised the findings of this work. All authors discussed the results and contributed to the final manuscript.

## Conflict of Interest

The authors declare that the research was conducted in the absence of any commercial or financial relationships that could be construed as a potential conflict of interest.
