# Supplementary material for: The Power of Feedback Revisited: A Meta-Analysis of Educational Feedback Research
Source: Front Psychol. 2020 Jan 22;10:3087. doi: 10.3389/fpsyg.2019.03087 (PMC6987456; doi:10.3389/fpsyg.2019.03087)
Supplement: Supplementary file 1 [file Data_Sheet_1.PDF]

Appendix

Studies used for meta-analysis

Adler, M. (1998). The effects of instruction in six trait writing on third grade students' writing abilities and attitudes toward writing (Unpublished master's thesis). Emporia State University, Emporia, KS.

Amabile, T. M., DeJong, W., & Lepper, M. R. (1976). Effects of externally imposed deadlines on subsequent intrinsic motivation. *Journal of Personality and Social Psychology, 34,* 92–98.

Ammar, A., & Spada, N. (2006). One size fits all? Recasts, prompts, and L2 learning. *Studies in Second Language Acquisition, 28,* 543-574. https://doi.org/10.1017/S0272263106060268

Anane, F. J. K. (1987). The delay-retention phenomena: The effect of differential instructions on the learning and remembering of prose text material. *Dissertation Abstracts International, 48, 2831A.* (University Microfilms No. 87–28,257)

Anderson, D. C., Crowell, C. R., Doman, M., & Howard, G. S. (1988). Performance posting, goal setting, and activity-contingent praise as applied to a university hockey team. *Journal of Applied Psychology, 73,* 87–95. https://doi.org/10.1037/0021–9010.73.1.87

Anderson, R. C., Kulhavy, R. W., & Andre, T. (1971). Feedback procedures in programmed instruction. *Journal of Educational Psychology, 62*(2), 148–156. https://doi.org/10.1037/h0030766

Anderson, R. C., Kulhavy, R. W., & Andre, T. (1972). Conditions under which feedback facilitates learning from programmed lessons. *Journal of Educational Psychology, 63*(3), 186–188. https://doi.org/10.1037/h0032653

Anshel, M. H. (1987). The effect of mood and pleasant versus unpleasant information feedback on performing a motor skill. *Journal of General Psychology, 115*, 117–129. https://doi.org/10.1080/00221309.1988.9711095

Arkin, R. M., & Schumann, D. W. (1984). Effects of corrective testing: An extension. *Journal of Educational Psychology, 76*, 835–843. https://doi.org/10.1037/0022-0663.76.5.835

Arkin, R. M., & Waits, E. A. (1983). Performance implications of corrective testing. *Journal of Educational Psychology, 75,* 561–57 1. https://doi.org/10.1037/0022-0663.75.4.561

Armour-Thomas, E., White, M. A., & Boehm, A. (1987). The motivational effects of eyes of feedback on children's learning concepts and retention of relational concepts. Paper presented at the Annual Meeting of the American Educational Research Association.

Arnett, P. P. (1985). Effects of feedback placement and completeness within Gagne's model for computer assisted instruction lesson development on concept and rule learning. *Dissertation Abstracts International, 46*, 2537A. (University Microfilms No. 85–24,309)

Arnone, M. P., & Grabowski, B. L. (1992). Effects on children's achievement and curiosity in learner control over an interactive video lesson. *Educational Technology Research & Development, 40*, 15–27. https://doi.org/10.1007/BF02296702

Arter, J. (1994). The impact of training students to be self-assessors of writing. Paper presented at the annual meeting of the American Educational Research Association, New Orleans.

Ashwell, T. (2000). Patterns of teacher response to student writing in a multiple-draft composition classroom: Is content feedback followed by form feedback the best method? *Journal of Second Language Writing, 9*, 227–257. https://doi.org/10.1016/S1060–3743(00)00027–8

Aumiller, L. E. (1963). The effects of knowledge of results on learning to spell new words by third and fifth grade pupils. *Dissertation Abstracts International, 24*, 5187. (University Microfilms No. 64–5336)

Austin, M. F., & Grant, T. N. (1981). Interview training for college students disadvantaged in the labor market: Comparison of five instructional techniques. *Journal of Counseling Psychology, 28*, 72–75. https://doi.org/10.1037/0022-0167.28.1.72

Ayoun, D. (2001). The role of negative and positive feedback in the second language acquisition of the passe' compose' and imparfait. *Modern Language Journal, 85*, 226–243. https://doi.org/10.1111/0026–7902.00106

Baechle, C. L., & Lian, M. J. (1990). The effects of direct feedback and practice on metaphor performance in children with learning disabilities. *Journal of Learning Disabilities, 23,* 451–455. https://doi.org/10.1177/002221949002300710

Bandura, A., & Cervone, D. (1983). Self-evaluative and self-efficacy mechanisms governing the motivational effects of goal systems. *Journal of Personality and Social Psychology, 45,* 1017–1028. https://doi.org/10.1037/0022–3514.45.5.1017

Baron, R. A. (1988). Negative effects of destructive criticism: Impact on conflict, self-efficacy and task performance. *Journal of Applied Psychology, 73*, 199–207. https://doi.org/10.1037/0021–9010.73.2.199

Bationo, B. (1991). The effects of three forms of immediate feedback on learning intellectual skills in a foreign language computer-based tutorial. Unpublished doctoral dissertation. The University of Toledo, Toledo, OH.

Battig, W. F., & Brackett, H. R. (1961). Comparison of anticipation and recall methods in paired-associate learning. *Psychological Reports, 9,* 59–65. https://doi.org/10.2466/pr0.1961.9.1.59

Bell-Corrales, M. (2001). The role of negative feedback in second language instruction. Unpublished doctoral dissertation. University of Florida, Gainesville.

Benson, J. S., & Yeany, R. H. (1986). Generalizability of diagnostic-prescriptive teaching strategies across student locus of control and multiple instructional units. *Journal of Research in Science Teaching, 23*(2), 113–120. https://doi.org/10.1002/tea.3660230204

Benson, N. (1979). The effects of peer feedback during the writing process on writing performance, revision behavior, and attitude toward writing (Unpublished doctoral dissertation). University of Colorado.

Berg, E. C. (1999). The effects of trained peer response on ESL students' revision types and writing quality. *Journal of second language writing, 8*(3), 215–241. https://doi.org/10.1016/S1060–3743(99)80115–5

Bethge, H.-J., Carlson, J. S., & Wiedl, K. H. (1982). The effects of dynamic assessment procedures on Raven Matrices performance, visual search behavior, test anxiety and test orientation. *Intelligence, 6*(1), 89–97. https://doi.org/10.1016/0160–2896(82)90022–8

Betz, N. E., & Weiss, D. J. (1976). Psychological Effects of Immediate Knowledge of Results and Adaptive Ability Testing. *Research Report 76–4*. https://doi.org/10.1037/e523172009-001

Bilodeau, E. A., & Ryan, F. J. (1960). A test for interaction of delay of knowledge of results and two types of interpolated activity. *Journal of Experimental Psychology, 59*, 414–419. https://doi.org/10.1037/h0048088

Bilsky, L. H., Gilbert, L., & Pawelski, C. E. (1978). Facilitation of class-inclusion performance in mildly retarded adolescents: Feedback and strategy training. *American Journal of Mental Deficiency*, 83, 177–184.

Bitchener, J. (1999). The negotiation of meaning by advanced ESL learners: The effects of individual learner factors and task type (Doctoral dissertation, ResearchSpace@ Auckland).

Bitchener, J. (2008). Evidence in support of written corrective feedback. *Journal of Second Language Writing, 17*, 102–118. https://doi.org/10.1016/j.jslw.2007.11.004

Bitchener, J., & Knoch, U. (2008). The value of written corrective feedback for migrant and international students. *Language teaching research, 12*(3), 409–431. https://doi.org/10.1177/1362168808089924

Bitchener, J., & Knoch, U. (2010). Raising the linguistic accuracy level of advanced L2 writers with written corrective feedback. *Journal of Second Language Writing*, *19*(4), 207–217. https://doi.org/10.1016/j.jslw.2010.10.002

Bitchener, J., Young, S., & Cameron, D. (2005). The effect of different types of corrective feedback on ESL student writing. *Journal of second language writing*, *14*(3), 191–205. https://doi.org/10.1016/j.jslw.2005.08.001

Bitchener, J., Young, S., & Cameron, D. (2005). The effect of different types of feedback on ESL student writing. *Journal of Second Language Writing, 14*, 191–205. https://doi.org/10.1016/j.jslw.2005.08.001

Boersma, F. J. (1966). Effects of delay of information feedback and length of postfeedback interval on linear programming. *Journal of Educational Psychology, 57*, 140–145. https://doi.org/10.1037/h0023405

Boggiano, A. K., & Barrett, M. (1985). Performance and motivational deficits of helplessness: The role of motivational orientations. *Journal of Personality and Social Psychology, 49*, 1753–176. https://doi.org/10.1037/0022–3514.49.6.1753

Boggiano, A. K., & Ruble, D. N. (1979). Competence and the overjustification effect: A developmental study. *Journal of Personality and Social Psychology, 37*(9), 1462. https://doi.org/10.1037/0022–3514.37.9.1462

Borland, J. H., & Wright, L. (1994). Identifying young, potentially gifted, economically disadvantaged students. *Gifted Child Quarterly, 38*, 164–171. https://doi.org/10.1177/001698629403800402

Boscolo, P., & Ascorti, K. (2004). Effects of collaborative revision on children's ability to write understandable narrative text. In L. Allal, L. Chanquoy, & P. Largy (Eds.), *Cognitive and instructional processes* (pp. 157–170). Boston: Kluwer. https://doi.org/10.1007/978–94-007–1048–1_10

Boulter, L. R. (1964). Evaluation of mechanisms in delay of knowledge of results. *Canadian Journal of Psychology, 18,* 281–291. https://doi.org/10.1037/h0083306

Bowles, M. A. (2005). Effects of verbalization condition and type of feedback on L2 development in a CALL task (Doctoral dissertation). Available from Proquest UMI Dissertation Publishing database. (Proquest No. 3175847)

Brabender, V., & Boardman, S. K. (1977). Sex differences in self-confidence as a function of feedback and social cues. *Psychological Reports, 41*(3), 1007–1010. https://doi.org/10.2466/pr0.1977.41.3.1007

Brabender, Y, & Boardman, S. K. (1977). Sex differences in self-confidence as a function of feedback and social cues. *Psychological Reports, 41,* 1007–1010. https://doi.org/10.2466/pr0.1977.41.3.1007

Brackbill, Y., & Kappy, M. S. (1962). Delay of reinforcement and retention. *Journal of Comparative and Physiological Psychology, 55*, 14–18. https://doi.org/10.1037/h0048741

Brakel Olson, V. L. (1990). The revising processes of sixth-grade writers with and without peer feedback. *Journal of Educational Research, 84*, 22–29. https://doi.org/10.1080/00220671.1990.10885987

Braunstein, D. N., Klein, G. A., & Paehla, M. (1973). Feedback expectancy and shifts in students' ratings of college faculty. *Journal of Applied Psychology, 58*. 254–258. https://doi.org/10.1037/h0035631

Bray, J. H., & Howard, G. S. (1980). Methodological considerations in the evaluation of teacher-training programs. *Journal of Educational Psychology. 72*, 62–70. https://doi.org/10.1037/0022-0663.72.1.62

Bridgeman, B. (1974). Effects of test score feedback on immediately subsequent test performance. *Journal of Educational Psychology, 66,* 62–66. https://doi.org/10.1037/h0035803

Brockner, J. & Vasta, R. (1981). Do causal attributions mediate the effects of extrinsic rewards on intrinsic interests? *Journal of Research in Personality, 15*, 201–209. https://doi.org/10.1016/0092–6566(81)90019–2

Brown, R. S. (1964). An experimental study of knowledge of results in complex human learning. *Dissertation Abstracts International, 24*, 2356. (University Microfilms No. 64–696).

Budoff, M., & Corman, L. (1974). Demographic and psychometric factors related to improved performance on the Kohs Learning-Potential Procedure. *American Journal of Mental Deficiency, 78,* 578–585.

Bumgarner, K. M. (1984). Effects of informational feedback and social reinforcement on elementary students' achievement during CAI drill and practice on multiplication facts. *Dissertation Abstracts International, 45*, 1102A. (University Microfilms No. 84–15,896)

Bustamante, J., Moreno, P., Rehbein, L., & Vizueta, A. (1980). Effects of feedback and reinforcement in tachistoscopic training on a fault-detection task. *Perceptual and Motor Skills, 51,* 987–993. https://doi.org/10.2466/pms.1980.51.3.987

Bustamante, J., Moreno, P., Rehbein, L., & Vizueta, A. (1980). Effects of feedback and reinforcement in tachistoscopic training on a fault-detection task. *Perceptual and Motor Skills, 51*(3), 987–993. https://doi.org/10.2466/pms.1980.51.3.987

Butler, R. (1987). Task-involving and ego-involving properties of evaluation: Effects of different feedback conditions on motivational perceptions, interest, and performance. *Journal of Educational Psychology, 79,* 474–482. https://doi.org/10.1037/0022-0663.79.4.474

Butler, R., & Nisan, M. (1986). Effects of no feedback, task-related comments, and grades on intrinsic motivation and performance. *Journal of Educational Psychology*, 78(3), 210. https://doi.org/10.1037/0022-0663.78.3.210

Caccamise, D., Franzke, M., Eckhoff, A., Kintsch, E., & Kintsch, W. (2007). Guided practice in technology-based summary writing. In D. S. McNamara (Ed.), *Reading comprehension strategies: Theory, interventions, and technologies*. Mahwah, NJ: Erlbaum.

Cameron, B., & Dwyer, F. (2005). The effect of online gaming, cognition and feedback type in facilitating delayed achievement of different learning objectives. *Journal of Interactive Learning Research, 16,* 243–258.

Cardelle, M., & Corno, L. (1981). Effects on second language learning of variations in written feedback on homework assignments. *TESOL Quarterly, 15*, 251–261. https://doi.org/10.2307/3586751

Carels, E. J. (1975). The effects of false feedback, sex, and personality on learning, retention, and the Zeigarnik effect in programmed instruction. *Dissertation Abstracts International, 36*, 2094A. (University Microfilms No. 75–22,345)

Carlson, J. S., & Wiedl, K. H. (1979). Toward a differential testing approach: Testing the limits employing the Raven Matrices. *Intelligence, 3,* 323–344. https://doi.org/10.1016/0160–2896(79)90002–3

Carroll, J. M., & Kay, D. S. (1988). Prompting, feedback and error correction in the design of a scenario machine. *International Journal of Man-Machine Studies, 28*(1), 11–27. https://doi.org/10.1016/S0020-7373(88)80050-6

Carroll, S., & Swain, M. (1993). Explicit and implicit negative feedback: An empirical study of the learning of linguistic generalization. *Studies in Second Language Acquisition, 15*, 357–386. https://doi.org/10.1017/S0272263100012158

Carroll, S., Swain, M., & Roberge, Y. (1992). The role of feedback in adult second language acquisition: Error correction and morphological generalizations. *Applied Psycholinguistics, 13*, 173–198. https://doi.org/10.1017/S0142716400005555

Carson, P. P., & Carson. K. D. (1993). Managing creativity enhancement through goal-setting and feedback. *Journal of Creative Behavior, 27*, 36–45. https://doi.org/10.1002/j.2162–6057.1993.tb01385.x

Carter, K. R. (1975). The effect of student feedback in modifying teaching performance. *Dissertation Abstracts International, 35*, 51l0A. (University Microfilms No. 75–2572)

Centra, J. A. (1973). Effectiveness of student feedback in modifying college instruction. *Journal of Educational Psychology. 65,* 395- 401. https://doi.org/10.1037/h0035636

Chandler, J. (2003). The efficacy of various kinds of error feedback for improvement in the accuracy and fluency of L2 student writing. *Journal of Second Language Writing, 12*, 267–296. https://doi.org/10.1016/S1060–3743(03)00038–9

Chanond, K. (1988). The effects of feedback, correctness of response and response confidence on learner's retention in computer-assisted instruction. Proceedings of selected research papers presented at the annual meetings of the Association for Educational Communications and Technology, New Orlans, LA.

Chen, H. J. H. (1996). A study of the effect of corrective feedback on foreign language learning: American students learning Chinese classifiers. Unpublished doctoral dissertation. University of Pennsylvania, Philadelphia.

Chhokar, J. S., & Wallin, J. A. (1984). A filed study of the effect of feedback frequency on performance. *Journal of Applied Psychology, 69*(3), 524–530. https://doi.org/10.1037/0021–9010.69.3.524

Chung, K. H., & Oean, V. (1976). Relative effectiveness and joint effects of three selected reinforcements in a repetitive task situation. *Organizational Behavior and Human Performance, 16,* 114–142. https://doi.org/10.1016/0030-5073(76)90009-X

Clariana, R. B., & Lee, D. (2001). The effects of recognition and recall study tasks with feedback in a computer-based vocabulary lesson. *Educational Technology Research and* Development, 49, 23–36. https://doi.org/10.1007/BF02504913

Clariana, R. B., Ross, S. M., & Morrison, G. R. (1991). The effects of different feedback strategies using computer-administered multiple-choice questions as instruction. *Educational Technology*, *Research and Development, 39*(2), 5–17. https://doi.org/10.1007/BF02298149

Clark, C. R., Guskey, T. R., & Benninga, J. S. (1983). The effectiveness of mastery learning strategies in undergraduate education courses. *Journal of Educational Research, 76*, 210–214.

Clodfelder, D. L. (1968). The quiz, knowledge of results, and individual differences in achievement orientation. Dissertation Abstracts International, 29, 2217. (University Microfilms No. 68–17181)

Coe, M., Hanita, M., Nishioka, V., & Smiley, R. (2011). An investigation of the impact of the 6 +1 Trait Writing model on grade 5 student writing achievement (NCEE 2012–4010). Washington, DC: National Center for Education Evaluation and Regional Assistance, Institute of Education Sciences, U.S. Department of Education.

Collins, M., Carnine, D., & Gersten, R. (1987). Elaborated corrective feedback and the acquisition of reasoning skills: A study of computer-assisted instruction. *Exceptional Children, 54*, 254–262. https://doi.org/10.1177/001440298705400308

Corbalan, G., Kester, L., & Van Merriënboer, J. J. G. (2009). Dynamic task selection: Effects of feedback and learner control on efficiency and motivation. *Learning and Instruction, 19,* 455–465. https://doi.org/10.1016/j.learninstruc.2008.07.002

Corbalan, G., Paas, F., & Cuypers, H. (2010). Computer-based feedback in linear algebra: Effects on transfer performance and motivation. *Computers & Education, 55*(2), 692–703. https://doi.org/10.1016/j.compedu.2010.03.002

Cormier, P., Carlson, J. S., & Das, J. P. (1990). Planning ability and cognitive performance: The compensatory effects of a dynamic assessment approach. *Learning and Individual Differences, 2,* 437–449. https://doi.org/10.1016/1041–6080(90)90004-Z

Daniel, T. L. & Esser, J. K. (1980). Intrinsic motivation as influenced by rewards, task interest, and task structure. *Journal of Applied Psychology, 65*, 566–573. https://doi.org/10.1037/0021–9010.65.5.566

Davis, W., & Fulton, J. (1997). The effects of professors' feedback on the growth of students' overall writing quality in two college freshman English courses. Retrieved from ERIC database. (ED414570)

Davis, W., & Mahoney, K. (1999). The Effects of Computer Skills and Feedback on the Gains in Students' Overall Writing Quality in College Freshman Composition Courses.

Day, J. D., & Cordon, L. A. (1993). Static and dynamic measures of ability: An experimental comparison. *Journal of Educational Psychology, 85,* 75–82. https://doi.org/10.1037/0022-0663.85.1.75

Day, J. D., & Cordón, L. A. (1993). Static and dynamic measures of ability: An experimental comparison. *Journal of Educational Psychology, 85*(1), 75. https://doi.org/10.1037/0022-0663.85.1.75

Deci, E. L. (1971). Effects of externally mediated rewards on intrinsic motivation. *Journal of Personality and Social Psychology*, 18, 105–115. https://doi.org/10.1037/h0030644

Deci, E. L., Betley, G., Kahle, J., Abrams, L., & Porac, J. (1981). When trying to win: Competition and intrinsic motivation. *Personality and Social Psychology Bulletin, 7,* 79–83. https://doi.org/10.1177/014616728171012

DeKeyser, R. (1993). The effect of error correction on L2 grammar knowledge and oral proficiency. *Modern Language Journal, 77,* 501–514. https://doi.org/10.1111/j.1540–4781.1993.tb01999.x

Dillashaw, F. G., & Okey, J. R. (1983). Effects of a modified mastery learning strategy on achievement, attitudes, and on-task behavior of high school chemistry students. *Journal of Research in Science Teaching, 20*(3), 203–211. https://doi.org/10.1002/tea.3660200304

Dollinger, S. J., & Thelen, M. H. (1978). Overjustification and children's intrinsic motivation: Comparative effects of four rewards. *Journal of Personality and Social Psychology, 36,* 1259–1269. https://doi.org/10.1037/0022–3514.36.11.1259

Dorow, L. G. (1976). Televised music lessons as educational reinforcement for correct mathematical responses with the educable mentally retarded. *Journal of Music Therapy, 11*, 77–86. https://doi.org/10.1093/jmt/13.2.77

Dossett, D. L., Latham, G. P., & Mitchell, T. R. (1979). Effects of assigned versus participatively set goals, knowledge of results, and individual differences on behavior when goal difficulty is held constant. *Journal of Applied Psychology, 64,* 291–298. https://doi.org/10.1037/0021–9010.64.3.291

Dyal, J. A. (1964). Effects of delay of knowledge of results in a line-drawing task. *Perceptual and Motor Skills*, 19(2), 433–434. https://doi.org/10.2466/pms.1964.19.2.433

Dyal, J. A., Wilson, W. J., & Berry, K. K. (1965). Acquisition and extinction of a simple motor skill as a function of delay of knowledge of results. Quarterly Journal *of Experimental Psychology, 17,* 158–162. https://doi.org/10.1080/17470216508416425

Earn, B. M. (1982). Intrinsic motivation as a function of extrinsic financial rewards and subjects' locus of control. *Journal of Personality, 50*, 360–373. https://doi.org/10.1111/j.1467–6494.1982.tb00756.x

Eisenstein, S. R. (1974). Effect of Contingent Guitar Lessons on Reading Behavior. *Journal of Music Therapy, 11*(3), 138–146. https://doi.org/10.1093/jmt/11.3.138

Elliot, B. A. (1986). An investigation of the effects of computer feedback and interspersed questions on the text comprehension of poor readers. *Dissertation Abstracts International, 47*, 2971A. (University Microfilms No. 86–27,446)

Ellis, R. (2007). The differential effects of corrective feedback on two grammatical structures. In A. Mackey (Ed.), *Conversational interaction in second language acquisition* (pp. 339–360). New York: Oxford University Press.

Ellis, R., Loewen, S., & Erlam, R. (2006). Implicit and explicit corrective feedback and the acquisition of L2 grammar. *Studies in Second Language Acquisition, 28*, 339–368. https://doi.org/10.1017/S0272263106060141

Ellis, R., Rosszell, H., & Takashima, H. (1994). Down the garden path: Another look at negative feedback. *JALT Journal, 16*, 9–24.

Ellis, R., Sheen, Y., Murakami, M., & Takashima, H. (2008). The effects of focused and unfocused written corrective feedback in an English as a foreign language context. *System, 36,* 353–371. https://doi.org/10.1016/j.system.2008.02.001

Embretson, S. E. (1987). Improving the measurement of spatial aptitude by dynamic testing. *Intelligence, 11*, 333–358. https://doi.org/10.1016/0160–2896(87)90016-X

Emmer, E., Mcburnette, P., & Davis, 0. (1974). Instructor perception, concern of scale, and feedback effectiveness. Paper presented at the annual meeting of the American Educational Research Association, Chicago. (ERIC Document Reproduction Service No. ED 103–399)

English, R. A., & Kinzer, J. R. (1966). The effect of immediate and delayed feedback on retention of subject matter. *Psychology in the Schools, 3*, 143–147. https://doi.org/10.1002/1520-6807(196604)3:2<143::AID-PITS2310030209>3.0.CO;2–1

Enzle, M. E., & Ross, J. M. (1978). Increasing and decreasing intrinsic interest with contingent rewards: A test of cognitive evaluation theory. *Journal of Experimental Social Psychology*, 14(6), 588–597. https://doi.org/10.1016/0022–1031(78)90052–5

Epstein, J. I. (1997). The effects of different types of feedback on learning verbal reasoning in computer-based instruction (Doctoral dissertation). Available from ProQuest Dissertations and Theses database. (UMI No. 9727464)

Erez, M. (1977). Feedback: A necessary condition for the goal setting-performance relationship. *Journal of Applied Psychology, 62*,624–627. https://doi.org/10.1037/0021–9010.62.5.624

Erickson, G. R., & Erickson, B. L. (1979). Improving college teaching: An evaluation of a teaching consultation procedure. *Journal of Higher Education, 50*, 670-683. https://doi.org/10.1080/00221546.1979.11780000

Erickson, G. R., & Sheehan, D. (1976). An evaluation of a teaching improvement process for university faculty. Paper presented at the annual meeting of the American Educational Research Association, San Francisco. (ERIC document Reproduction Service No. ED 131 111)

Evans, N. W., Hartshorn, K. J., & Strong-Krause, D. (2011). The efficacy of dynamic written corrective feedback for university-matriculated ESL learners. *System, 39*, 229–239. https://doi.org/10.1016/j.system.2011.04.012

Farr, J. L. (1976). Task characteristics, reward contingency, and intrinsic motivation. *Organizational Behavior and Human Performance, 16,* 294–307. https://doi.org/10.1016/0030-5073(76)90018-0

Farragher, P., & Szabo, M. (1986). Learning environmental science from text aided by a diagnostic and prescriptive instructional strategy. *Journal of Research in Science Teaching, 23*(6), 557–569. https://doi.org/10.1002/tea.3660230608

Fathman, A., & Whalley, E. (1990). Teacher response to student writing: Focus on form versus content. In B. Kroll (Ed.), *Second language writing: Research insights for the classroom* (pp. 178–190). Cambridge, UK: Cambridge University Press. https://doi.org/10.1017/CBO9781139524551.016

Fazio, L. (2001). The effect of corrections and commentaries on the journal writing accuracy of minority- and majority-language students. *Journal of Second Language Writing, 10*, 235–249. https://doi.org/10.1016/S1060–3743(01)00042-X

Feldhusen, J. F., & Birt, A. (1962). A study of nine methods of presentation of programmed learning material. *The Journal of Educational Research, 5*, 461–466. https://doi.org/10.1080/00220671.1962.10882854

Ferris, D. (2006). Does error feedback help student writers? New evidence on the short- and long-term effects of written error correction. In K. Hyland & F. Hyland (Eds.), *Feedback in second language writing: Contexts and issues* (pp. 81–105). Cambridge, UK: Cambridge University Press. https://doi.org/10.1017/CBO9781139524742.007

Ferris, D., & Roberts, B. (2001). Error feedback in L2 writing classes: How explicit does it need to be?. *Journal of second language writing, 10*(3), 161–184. https://doi.org/10.1016/S1060–3743(01)00039-X

Franzke, M., Kintsch, E., Caccamise, D., Johnson, N., & Dooley, S. (2005). Summary Street: Computer support for comprehension and writing. *Journal of Educational Computing Research, 33*, 53- 80. https://doi.org/10.2190/DH8F-QJWM-J457-FQVB

Froman, R. D. & Owen, S. V. (1980 Influence of different types of student ratings feedback upon later instructional behavior. Paper presented at the annual meeting of the American Educational Research Association, Boston. (ERIC Document Reproduction Service No. ED 187 724)

Fuchs, L. S., Fuchs, D., & Hamlett, C. L. (1989). Computers and curriculum-based measurement: Teacher feedback systems. *School Psychology Review, 18*, 112–125.

Fuchs, L. S., Fuchs, D., Hamlett, C. L., & Allinder, R. M. (1991a). Effects of expert system advice within curriculum-based measurement on teacher planning and student achievement in spelling. *School Psychology Review, 20*, 49 - 66.

Fuchs, L. S., Fuchs, D., Hamlett, C. L., & Allinder, R. M. (1991b). The contribution of skills analysis to curriculum-based measurement in spelling. *Exceptional Children, 57*, 443 https://doi.org/10.1177/001440299105700507

Fukuya, J. Y., & Zhang, Y. (2002). Effects of recasts on EFL learners' acquisition of pragmalinguistic conventions of request. *Second Language Studies, 21*, 1–47.

Fulmer, R. S., & Rollings, H. E. (1976). Item-by-item feedback and multiple-choice test performance. *Journal of Experimental Education, 44*, 30–32. https://doi.org/10.1080/00220973.1976.11011546

Gaynor, P. (1981). The effect of feedback delay on retention of computer-based mathematical material. *Journal of Computer-Based Instruction, 8*, 28–34.

Geen, R. G. (1981). Effects of being observed on persistence at an insolvable task. *British Journal of social Psychology, 20,* 211–216. https://doi.org/10.1111/j.2044–8309.1981.tb00534.x

Gherfal, I. R. (1982). The application of principles of reinforcement to the teaching of English as a second language in a developing country: an experiment with Libyan male preparatory school students from culturally diverse rural and urban communities. Dissertation Abstracts International, 43, 147A. (University Microfilms No. 82–13,750)

Gilman, D. A. (1969). Comparison of several feedback methods for correcting errors by computer-assisted instruction. *Journal of Educational Psychology, 60*, 503-508. https://doi.org/10.1037/h0028501

Glover, J. A. (1989). Improving readers' estimates of learning from text: The role of inserted questions. *Reading Research and Instruction, 28*, 68–75. https://doi.org/10.1080/19388078909557976

Goltz, S. M. (1990). Investigating self, task and supervisory feedback effects (Research paper Series 90-01). Notre Dame, IN: University of Notre Dame, Center for Research in Business.

Gordijn, J., & Nijhof, W. J. (2002). Effects of complex feedback on computer-assisted modular instruction. *Computers and Education, 39*, 183–200. https://doi.org/10.1016/S0360–1315(02)00025–8

Gordon, M. V. (1977). The effects of contingent instrumental music instruction on the language reading behavior and musical performance ability of middle school students. Unpublished doctoral dissertation, Columbia University Teachers College.

Greene, D., & Lepper, M. R. (1974). Effects of extrinsic rewards on children's subsequent intrinsic interest. *Child Development, 45*, 1141–1145. https://doi.org/10.2307/1128110

Greer, R. D., Randall, A., & Timberlake, C. (1971). The discriminate use of music listening as a contingency for improvement in vocal pitch acuity and attending behavior. Bulletin of the Council for Research in Music Education, 26, 10–18.

Guastello, E. F. (2001). Parents as partners: Improving children's writing. Celebrating the Voices of Literacy: The Twenty-Third Yearbook of the College Reading Association: A Peer Reviewed Publication of the College Reading Association, 279–293.

Hall, K. A., Adams, M., & Tardibuono, J. (1968). Gradient- and full-response feedback in computer-assisted instruction. *Journal of Educational Research, 61*, 195–199. https://doi.org/10.1080/00220671.1968.10883643

Hamner, W. C. & Eoster, L. (1975). Are intrinsic and extrinsic rewards additive: A test of Deci's cognitive evaluation theory of task motivation. *Organizational Behavior and Human Performance, 14,* 398–415. https://doi.org/10.1016/0030-5073(75)90038-0

Han, Z. (2002). A study of the impact of recasts on tense consistency in L2 output. *TESOL Quarterly, 36,* 543-572. https://doi.org/10.2307/3588240

Hanna, G. S. (1976). Effects of total and partial feedback in multiple-choice testing upon learning. *Journal of Educational Research, 69*, 202–205. https://doi.org/10.1080/00220671.1976.10884873

Harackiewicz, J. M. (1979). The effects of reward contingency and performance feedback on intrinsic motivation. *Journal of Personality and Social Psychology, 37*, 1352–1363. https://doi.org/10.1037/0022–3514.37.8.1352

Harackiewicz, J. M., Manderlink, G., & Sansone, C. (1984). Rewarding pinball wizardry: Effects of evaluation and cue value on intrinsic interest. *Journal of Personality and Social Psychology, 47*(2), 287–300. https://doi.org/10.1037/0022–3514.47.2.287

Hartshorn, K. J., Evans, N. W., Merrill, P. F., Sudweeks, R. R., Strong-Krause, D., & Anderson, N. J. (2010). Effects of dynamic corrective feedback on ESL writing accuracy. *TESOL Quarterly, 44,* 84–109. https://doi.org/10.5054/tq.2010.213781

Heald, H. M. (1970). The effects of immediate knowledge of results and correlation of errors and text anxiety upon test performance. *Dissertation Abstracts International, 31*, 1621A. (University Microfilms No. 70–17,724)

Herron, C. (1991). The garden path correction strategy in the foreign language classroom. *The French Review, 64,* 966–977.

Herron, C., & Tomasello, M. (1988). Learning grammatical structures in a foreign language: Modeling versus feedback. *The French Review, 61,* 910–922.

Hillocks, G. (1982). The interaction of instruction, teacher comment, and revision in teaching the composing process. *Research in the Teaching of English, 16,* 261–277.

Hines, S. J., & Seidman, S. A. (1988, January). The effects of selected CAI design strategies on achievement, and an exploration of other related factors. Proceedings of selected research papers presented at the annual meeting of the Association for Educational Communications and Technology, New Orleans, LA.

Hines, S. J., & Seidman, S. A. (1988). The effects of selected CAI design strategies on achievement, and an exploration of other related factors. Proceedings of selected research papers presented at the annual meeting of the Association for Educational Communications and Technology, New Orleans, LA.

Hino, J. (2006). Linguistic information supplied by negative feedback: A study of its contribution to the process of second language acquisition. Unpublished doctoral dissertation. University of Pennsylvania, Philadelphia.

Hodes, C. L. (1985). Relative effectiveness of corrective and noncorrective feedback in computer-assisted instruction on learning and achievement. *Journal of Educational Technology Systems, 13*, 249–254. https://doi.org/10.2190/D1PA-23WW-VHX1–7P52

Hoffman, B. S. (1974). An examination of knowledge of results and step size in programmed instruction at high and low cognitive levels of objectives. *Dissertation Abstracts International, 35*, 6006A. (University Microfilms No. 75-01,707)

Holliway, D. R. (2004). Through the eyes of my reader: A strategy for improving audience perspective in children's descriptive writing. *Journal of Research in Childhood Education, 18,* 334–349. https://doi.org/10.1080/02568540409595045

Holman, L. (2011). Automated writing evaluation program's effects on student writing achievement (Unpublished doctoral dissertation). Tennessee State University, Nashville.

Hough, J. B., & Revsin, B. (1963). Programmed instruction at the college level: A study of several factors influencing learning. *Phi Delta Kappan, 44*, 286–291.

Hoyt, D. P., & Howard, G. S. (1978). The evaluation of faculty development programs. *Research in Higher Education, 8,* 25–38. https://doi.org/10.1007/BF00985854

Huang, T. O. (2008). The role of task-specific adapted knowledge of response feed- back in algebra problem solving online homework in a college remedial course (Doctoral dissertation). Available from ProQuest Dissertations and Theses database. (UMI No. 3325192)

Hyman, C., & Tobias, S. (1981). Feedback and prior achievement. Paper presented at the annual meeting of the Northeastern Educational Research Association, Ellenville, NY. (ERIC Document Reproduction Service No. ED 206 739)

Hymel, G. M., & Mathews, G. S. (1980). A mastery approach to teaching U.S. history: The impact on cognitive achievement and unit evaluation. Paper presented at the annual meeting of the American Educational Research Association, Boston. (ERIC Document Reproduction Service No. ED 184 929)

Ifenthaler, D. (2010). Bridging the gap between expert-novice differences: The model-based feedback approach. *Journal of Research on Technology in Education, 43*, 103–117. https://doi.org/10.1080/15391523.2010.10782564

Ivancevich, J. M. (1982). Subordinates' reactions to performance appraisal interviews: A test of feedback and goal-setting techniques. *Journal of Applied Psychology, 67*, 581 -587. https://doi.org/10.1037/0021–9010.67.5.581

Ivancevich, J. M., & McMahon, J. T. (1982). The effects of goal setting, external feedback, and self-generated feedback on outcome variables: A field experiment. *Academy of Management Journal*, 25(2), 359–372. https://doi.org/10.5465/255997

Jacobs, P. I., & Kulkarni, S. (1966). A test of some assumptions underlying programmed instruction. *Psychological Reports, 18*, 103–110. https://doi.org/10.2466/pr0.1966.18.1.103

Jewell, J. (2003). The utility of curriculum-based measurement writing indices for progress monitoring and intervention (Unpublished doctoral dissertation). Northern Illinois University, Dekalb.

Jhowry, K. (2010). Does the provision of an intensive and highly focused indirect corrective feedback lead to accuracy? (Unpublished MA thesis). University of North Texas, Denton, TX.

Johansen, K. J., & Tennyson, R. D. (1983). Effect of adaptive advisement on perception in linear-controlled, computer-based instruction using a rule-learning task. *Educational Communication and Technology Journal, 31*, 226–236.

Johnson, J. M., & Zinner, C. C. (1974). Stimulus fading and scheduled learning in generalizing and maintaining behaviors. *Journal of Music Therapy, 11*, 80–96. https://doi.org/10.1093/jmt/11.2.84

Johnston, J. (1979). A comparison of mid-semester and end-of-semester student evaluation of faculty, Dissertation Abstracts International. 39, 6488A. (University Microfilms No. 79–10473)

Kamimura, T. (2006). Effects of peer feedback on ESL student writers at different levels of proficiency: A Japanese context. TESL Canada Journal, 23, 12–39. https://doi.org/10.18806/tesl.v23i2.53

Kang, H. (2007). Negative evidence: Its positioning, explicitness and linguistic focus as factors in second language acquisition. Unpublished doctoral dissertation. University of Pennsylvania, Philadelphia.

Kar, B. C., Dash, U. N., Das, J. P., & Carlson, J. S. (1993). Two experiments on the dynamic assessment of planning. *Learning and Individual Differences, 5,* 13–29. https://doi.org/10.1016/1041–6080(93)90023-L

Karraker, R. J. (1967). Knowledge of results and incorrect recall of plausible multiple-choice alternatives. *Journal of Educational Psychology, 58*, 11–14. https://doi.org/10.1037/h0024113

Keane, K. J., & Kretschmer, R. E. (1987). Effect of mediated learning intervention on cognitive task performance with a deaf population. *Journal of Educational Psychology, 79,* 49–53. https://doi.org/10.1037/0022-0663.79.1.49

Kellams, S. E., & Bohm, B. H. (1985). Are student ratings useful for improving the instruction of part-time faculty members. Paper presented at the annual meeting of the American Educational Research Association, Chicago.

Kepner, C. G. (1991). An experiment in the relationship of types of written feedback to the development of second-language writing skills. *Modern Language Journal, 75*, 305–313. https://doi.org/10.1111/j.1540–4781.1991.tb05359.x

Kim, J. S., & Hamner, W. C. (1976). Effect of performance feedback and goal setting on productivity and satisfaction in an organizational setting. *Journal of Applied Psychology, 61*(1), 48. https://doi.org/10.1037/0021–9010.61.1.48

Kim, J. Y. L., & Phillips, T. L. (1991). The effectiveness of two forms of corrective feedback in diabetes education. *Journal of Computer-Based Instruction, 18*, 14–18.

Kistner, J. A. (1985). Attentional deficit of learning-disabled children: Effects of rewards and practice. *Journal of Abnormal Child Psychology, 13*, 19–31. https://doi.org/10.1007/BF00918369

Koch, C. G., & Dorfman, P. W. (1979). Recall. And Recognition Processes in Motor Memory: Effects of Feedback and Knowledge of Results Delay. *Journal of Motor Behavior, 11*(1), 23–34. https://doi.org/10.1080/00222895.1979.10735169

Komaki, J. L., Collins, R. L., & Penn, P. (1982). The role of performance antecedents and consequences in work motivation. *Journal of Applied Psychology, 67*, 334–340. https://doi.org/10.1037/0021–9010.67.3.334

Komaki, J., Heinzmann, A. T., & Lawson, L. (1980). Effect of training and feedback: component analysis of a behavioral safety program. *Journal of applied Psychology, 65*(3), 261. https://doi.org/10.1037/0021–9010.65.3.261

Kopp, V., Stark, R., & Fischer, M. R. (2008). Fostering diagnostic knowledge through computer-supported, case-based worked examples: Effects of erroneous examples and feedback. *Medical Education, 42,* 823–829. https://doi.org/10.1111/j.1365–2923.2008.03122.x

Kozlow, M., & Bellamy, P. (2004). Experimental study on the impact of the 6 + 1 Trait Writing model on student achievement in writing. Portland, OR: Northwest Regional Educational Laboratory. Langer, J. (2011). Envisioning knowledge: Building literacy in the academic disciplines. New York: Teachers College Press.

Kramarski, B., & Zeichner, O. (2001). Using technology to enhance mathematical reasoning: Effects of feedback and self-regulation learning. *Educational Media International, 38*, 77–82. https://doi.org/10.1080/09523980110041458

Kratochwill, T. R., & Brody, G. H. (1976). Effects of verbal and self-monitoring feedback on Wechsler Adult Intelligence Scale performance in normal adults. *Journal of Consulting and Clinical Psychology, 44*(5), 879. https://doi.org/10.1037/0022-006X.44.5.879

Kruglanski, A. W., Alon, S., & Lewis, T. (1972). Retrospective misattribution and task enjoyment. *Journal of Experimental Social Psychology, 8,* 493-501. https://doi.org/10.1016/0022–1031(72)90075–3

Krumboltz, J. D., & Weisman, R. G. (1962). The effect of intermittent confirmation in programmed instruction. *Journal of Educational Psychology, 53*, 250–253. https://doi.org/10.1037/h0040448

Kubota, M. (1994). The role of negative feedback on the acquisition of the English dative alternation by Japanese college student of EFL. *Institute for Research in Language Teaching Bulletin, 8,* 1–36.

Kulhavy, R. W. (1977). Feedback in written instruction. Review of Educational Research, 47, 211 -232. https://doi.org/10.3102/00346543047002211

Kulhavy, R. W., Yekovich, F. R., & Dyer, J. W. (1976). Feedback and response confidence. *Journal of Educational Psychology, 68,* 522–528. https://doi.org/10.1037/0022-0663.68.5.522

Kunda, Z., & Schwartz, S. H. (1983). Undermining intrinsic moral motivation: External reward and self-presentation. *Journal of Personality and Social Psychology, 45*(4), 763. https://doi.org/10.1037/0022–3514.45.4.763

Lacher, M. B. (1983). Effects of feedback, instruction, and initial performance level upon training and persistence of verbal rehearsal. *Journal of General Psychology, 108*, 43-54. https://doi.org/10.1080/00221309.1983.9711477

Lacher, M. B. (1983). Effects of feedback, instruction, and initial performance level upon training and persistence of verbal rehearsal. *The Journal of General Psychology, 108*(1), 43-54. https://doi.org/10.1080/00221309.1983.9711477

Lalande, J. F. (1982). Reducing composition errors: An experiment. *Modern Language Journal, 66,* 140–149. https://doi.org/10.1111/j.1540–4781.1982.tb06973.x

Landsman, H. J., & Turkewitz, M. (1962). Delay of knowledge of results and performance on a cognitive task. *Psychological Reports, 11*, 66–70. https://doi.org/10.2466/pr0.1962.11.1.66

Larson, G. E., Alderton, D. L., & Kaupp, M. A. (1991). Dynamic administration of a general intelligence test. *Learning and Individual Differences, 3,* 123–134. https://doi.org/10.1016/1041–6080(91)90012-P

Larson, J. R., Jr., & Callahan, C. (1990). Performance monitoring: How it affects work productivity. *Journal of Applied Psychology, 75*, 530–538. https://doi.org/10.1037/0021–9010.75.5.530

Latta, R. M. (1978). Interactive effects of initial achievement orientation and prior success feedback on the mastery of subsequent difficult and easy tasks. *American Educational Research Journal, 15*, 17–24. https://doi.org/10.3102/00028312015001017

Lee, H. W., Lim, K. Y., & Grabowski, B. L. (2010). Improving self-regulation, learning strategy use, and achievement with metacognitive feedback. *Educational Technology Research and Development, 58,* 629–648. https://doi.org/10.1007/s11423-010–9153–6

Lee, I. (1997). ESL learners' performance in error correction in writing: Some implications for teaching. *System, 25*(4), 465–477. https://doi.org/10.1016/S0346–251X(97)00045–6

Leeman, J. (2003). Recasts and second language development: Beyond negative evidence. *Studies in Second Language Acquisition, 25*, 37–63. https://doi.org/10.1017/S0272263103000020

Leherissey, B. L., O'Neil, H. F., & Hansen, D. N. (1970). Effects of memory support on state anxiety and performance in computer-assisted learning (Contract No. N000I4–68-A-0494). Washington, DC: U.S. Office of Naval Research, Psychological Sciences Division, Personnel & Training Research Programs.

Lepper, M. R., Greene, D., & Nisbett, R. E. (1973). Undermining children's intrinsic interest with extrinsic rewards: A test of the "overjustification" hypothesis. *Journal of Personality and Social Psychology, 28,* 129–137. https://doi.org/10.1037/h0035519

Levine, F. M., Broderick, J. E., & Burkart, M. (1983). Attribution and contrast: Two explanations for the effects of external rewards on intrinsic motivation. *British Journal of Psychology, 74*(4), 461–466. https://doi.org/10.1111/j.2044–8295.1983.tb01878.x

Levy, J. (1974). Social reinforcement and knowledge of results as determinants of motor performance among EMR children. American Journal of Mental Deficiency. 1–16.

Lewis-Beck, J. A. (1978). Locus of control, task expectancies, and children's performance following failure. *Journal of Educational Research. 71*, 207–210. https://doi.org/10.1080/00220671.1978.10885072

Lhyle, K. G., & Kulhavy, R. W. (1987). Feedback processing and error correction. *Journal of Educational Psychology, 79,* 320–322. https://doi.org/10.1037/0022-0663.79.3.320

Lin, H. (2006). The effect of questions and feedback used to complement static and animated visualization on tests measuring different educational objectives (Doctoral dissertation). Available from ProQuest Dissertations and Theses database. (UMI No. 3318901)

Lipnevich, A. A., & Smith, J. K. (2009). Effects of differential feedback on students' examination performance. Journal of Experimental Psychology. *Journal of Applied Psychology, 15,* 319–333. https://doi.org/10.1037/a0017841

Locke, E. A. (1967). The motivational effects of knowledge of results: Knowledge or goal setting? *Journal of Applied Psychology,51*, 324–329 https://doi.org/10.1037/h0024771

Locke, E. A., & Bryan, J. F. (1968). Goal-setting as a determinant of the effect of knowledge of score on performance. *American Journal of Psychology, 79*, 398–406. https://doi.org/10.2307/1420637

Locke, E. A., & Shaw, K. N. (1984). Atkinson's inverse U curve and the missing cognitive variables. *Psychological Reports, 55,* 403–412. https://doi.org/10.2466/pr0.1984.55.2.403

Loewen, S., & Erlam, R. (2006). Corrective feedback in the chatroom: An experimental study. *Computer Assisted Language Learning, 19,* 1–14. https://doi.org/10.1080/09588220600803311

Loewen, S., & Nabei, T. (2007). Measuring the effects of oral corrective feedback on L2 knowledge. In A. Mackey (Ed.), *Conversational interaction in second language acquisition* (pp. 361–377). New York: Oxford University Press.

Long, J. C., Okey, J. R., & Yeany, R. H. (1978). The effects of diagnosis with teacher-or student-directed remediation on science achievement and attitudes. *Journal of Research in Science Teaching*, 15(6), 505–511. https://doi.org/10.1002/tea.3660150610

Long, M. H., Inagaki, S., & Ortega, L. (1998). The role of implicit negative feedback in SLA: Models and recasts in Japanese and Spanish. *The Modern Language Journal,* 82, 357–371. https://doi.org/10.1111/j.1540–4781.1998.tb01213.x

Lublin, S. C. (1965). Reinforcement schedules, scholastic aptitude, autonomy need, and achievement in a programed course. *Journal of Educational Psychology, 56*, 295–302. https://doi.org/10.1037/h0022790

Lumbelli, L., Paoletti, G., & Frausin, T. (1999). Improving the ability to detect comprehension problems: From revising to writing. *Learning and Instruction, 9*, 143–166. https://doi.org/10.1016/S0959–4752(98)00041–3

Luyten, H., & Lens, W. (1981). The effect of earlier experience and reward contingencies on intrinsic motivation. *Motivation and Emotion, 5*, 25–36. https://doi.org/10.1007/BF00993659

Lyster, R. (2004). Different effects of prompts and effects in form-focused instruction. Studies in Second *Language Acquisition, 26,* 399–432. https://doi.org/10.1017/S0272263104263021

MacArthur, C. A., Schwartz, S. S., & Graham, S. (1991). Effects of a reciprocal peer revision strategy in special education classrooms. *Learning Disabilities Research, 6,* 201–210.

Macheak, T. (2002). Learner vs. instructor correction in adult second language acquisition: Effects of oral feedback type on the learning of French grammar. *Dissertation Abstracts International, 64,* 2467.

Mackey, A., & Oliver, R. (2002). Interactional feedback and children's L2 development. *System, 30*, 459–477. https://doi.org/10.1016/S0346–251X(02)00049-0

Mackey, A., & Philp, J. (1998). Conversational Interaction and Second Language Development: Recasts, Responses, and Red Herrings? The Modern Language Journal, 82(3), 338–356. https://doi.org/10.1111/j.1540-4781.1998.tb01211.x

Madsen, C. K. (1981) . Music lessons and books as reinforcement alternatives for an academic task. *Journal of Research in Music Education, 29*, 100–110. https://doi.org/10.2307/3345019

Madsen, C. K. (1981). Music Lessons and Books as Reinforcement Alternatives for an Academic Task. *Journal of Research in Music Education, 29*(2), 103–110. https://doi.org/10.2307/3345019

Madsen, C. K., & Forsythe, J. L. (1973). Effect of contingent music listening on increases of mathematical responses. *Journal of Research in Music Education, 21*(2), 176–181. https://doi.org/10.2307/3344594

Madsen, C. K., & Geringer, J. (1976). Choice of televised music lessons versus free play in relationship to academic improvement. *Journal of Music Therapy, 19*, 154–162. https://doi.org/10.1093/jmt/13.4.154

Madsen, C. K., & Madsen, C. H. J r. (1972). Selection of music listening or candy as a function of contingent versus noncontingent reinforcement and scale singing. *Journal of Music Therapy, 9,* 190–198. https://doi.org/10.1093/jmt/9.4.190

Madsen, C. K., Dorow, L., Moore, R., & Womble, J. (1976). Effect of music via television as reinforcement for correct mathematics. *Journal of Research in Music Education, 24*, 51–59. https://doi.org/10.2307/3344936

Madsen, C. K., Moore, R. S., Wagner, M. J., & Yarbrough, C. (1975). A Comparison of Music as Reinforcement for Correct Mathematical Responses Versus Music as Reinforcement for Attentiveness. *Journal of Music Therapy, 12*(2), 84–95. https://doi.org/10.1093/jmt/12.2.84

Maheady, L., Harper, G. R., & Sacca, K. (1988). Classwide peer tutoring system in a secondary re- source program for the mildly handicapped. *Journal of Research and Development in Education, 21*(3), 76–83.

Manzer, C. W. (1935). The effect of knowledge of output on muscular work. *Journal of Experimental Psychology*, 18(1), 80. https://doi.org/10.1037/h0057862

Matsumura, L. C., Garnier, H., Pascal, J., & Valdes, R. (2002). Measuring Instructional Quality in Accountability Systems: Classroom Assignments and Student Achievement. *Educational Assessment, 8*(3), 207–229. https://doi.org/10.1207/S15326977EA0803_01

Matsumura, L. C., Patthey-Chavez, G. G., Valdes, R., & Garnier, H. (2002). Teacher feedback, writing assignment quality, and third-grade students' revision in lower- and higher-achieving urban schools. *Elementary School Journal, 103*, 3–22. https://doi.org/10.1086/499713

Mazingo, D. E. (2006). Identifying the relationship between feedback provided in computer-assisted instructional modules, science self-efficacy, and academic achievement (Doctoral dissertation). Available from ProQuest Dissertations and Theses database. (UMI No. 3263234)

McClintock, C. G., & Van Avermael, E. (1975). The effects of manipulating feedback upon children's motives and performance: A propositional statement and empirical evaluation. *Behavioral Science, 20*, 101–116. https://doi.org/10.1002/bs.3830200203

McCutchen, D., Francis, M., & Kerr, S. (1997). Revising for meaning: Effects of knowledge and strategy. *Journal of Educational Psychology, 89*, 667–676. https://doi.org/10.1037/0022-0663.89.4.667

McDonough, K. (2005). Identifying the impact of negative feedback and learners' response on ESL question development. *Studies in Second Language Acquisition, 27,* 79–103. https://doi.org/10.1017/S0272263105050047

McDonough, K. (2007). Interactional feedback and the emergence of simple past activity verbs in L2 English. In A. Mackey (Ed.), *Conversational interaction in second language acquisition* (pp. 323–338). New York: Oxford University Press.

McFarlin, D. B., Baumeister, R. F., & Blascovich, J. (1984). On knowing when to quit: Task failure, self-esteem, advice, and nonproductive persistence. *Journal of Personality, 52*, 138–155. https://doi.org/10.1111/j.1467–6494.1984.tb00349.x

McGroarty, M. E., & Zhu, W. (1997). Triangulation in classroom research: A study of peer revision. *Language Learning, 47,* 1–43. https://doi.org/10.1111/0023–8333.11997001

McKeachie, W. J., Lin, Y. G., Daugherty, M., Moffett, M. M., Neigler, C., Nork, J., & Baldwin, R. (1980). Using student ratings and consultation to improve instruction. *British Journal of Educational Psychology, 50*(2), 168–174. https://doi.org/10.1111/j.2044–8279.1980.tb02442.x

McKeachie, W., & Lin, Y. G. (1975). Use of student rulings in evaluation of college teaching. (Final report). Ann Arbor, MI: University of Michigan. (ERIC Document Reproduction Service No. ED 0fl6 411)

McLaughlin, T. F., & Helm, J. L. (1993). Use of contingent music to increase academic performance of middle-school students. *Psychological Reports, 72*(2), 658–658. https://doi.org/10.2466/pr0.1993.72.2.658

McLean, D. Y. (1979). The effects of midsemester feedback on weekly evaluations of university instructors. Unpublished roaster's thesis, University of Western Ontario, London, Canada.

Merril, J. (1987). Levels of questioning and forms of feedback: Instructional factors in courseware design. *Journal of Computer-Based Instruction, 14*, 18–22.

Mesch, D. J., Farh, 1., & Podsakoff, P. M. (1989). Effects of feedback sign on group goal setting, strategies, and performance: An empirical examination of some control theory hypotheses. Unpublished manuscript, Indiana University, Bloomington.

Meyer, E., Abrami, P., Wade, C., Aslan, O., & Deault, L. (2010). Improving literacy and metacognition with electronic portfolios: Teaching and learning with ePearl. *Computers & Education, 55,* 84 -91. https://doi.org/10.1016/j.compedu.2009.12.005

Miller, D. (1977). Effects of music listening contingencies on arithmetic performance amid music preference of EMR children. *American Journal of Mental Deficiency, 81*, 371–378.

Miller, D. M., Dorow, L., & Greer, R. D. (1974). The contingent use of music and art for improving arithmetic scores. *Journal of Music Therapy, 11,* 57–64. https://doi.org/10.1093/jmt/11.2.57

Miller, M. T. (1971). Instructor attitudes toward, and their use of, student ratings of teaches. *Journal of Educational Psychology, 62*, 235–239. https://doi.org/10.1037/h0031143

Miller, P. C. (2004). The effect of recasts on the acquisition of French: The case for the passe' compose'. Unpublished manuscript.

Missiuna, C., & Samuels, M. T. (1989). Dynamic assessment of preschool children with special needs: Comparison of mediation and instruction. *Remedial and Special Education, 10*, 53–62. https://doi.org/10.1177/074193258901000210

Moore, J. W., & Smith, W. I. (1961). Knowledge of results in self-teaching spelling. *Psychological Reports, 9,* 717–726. https://doi.org/10.2466/pr0.1961.9.3.717

More, A. J. (1969). Delay of feedback and the acquisition and retention of verbal materials in the classroom. *Journal of Educational Psychology, 60*, 339–342. https://doi.org/10.1037/h0028318

Moreno, N. (2007). The effects of type of task and type of feedback on L2 development in call (Doctoral dissertation). Available from ProQuest Dissertations and Theses database. (UMI No. 3302088)

Moreno, R. (2004). Decreasing Cognitive Load for Novice Students: Effects of Explanatory versus Corrective Feedback in Discovery-Based Multimedia. *Instructional Science, 32*(1/2), 99–113. https://doi.org/10.1023/B:TRUC.0000021811.66966.1d

Morrison, G. R., Ross, S. M., Gopalakrishnan, M., & Casey, J. (1995). The effects of feedback and incentives on achievement in computer-based instruction. *Contemporary Educational Psychology, 20*, 32–50. https://doi.org/10.1006/ceps.1995.1002

Mossholder, K. W. (1980). Effects of externally mediated goal setting on intrinsic motivation: A laboratory experiment. *Journal of Applied Psychology, 65*, 202–210. https://doi.org/10.1037/0021–9010.65.2.202

Munyofu, M. (2008). Effects of varied enhancement strategies (chunking, feedback, gaming) in complementing animated instruction in facilitating different types of learning objectives (Doctoral dissertation). Available from ProQuest Dissertations and Theses database. (UMI No. 3414357)

Muranoi, H. (2000). Focus on form through interaction enhancement: Integrating formal instruction into a communicative task in EFL classrooms. *Language Learning, 50*, 617–673. https://doi.org/10.1111/0023–8333.00142

Murphy, J. B., & Appel, V. H. (1978). The effects of mid-semester student feedback on instructional change and improvement. Paper presented at the annual meeting of the American Educational Research Association, Toronto.

Murphy, P. (2007). Reading comprehension exercises online: The effects of feedback, proficiency and interaction. *Language Learning & Technology, 11*(3), 107–129.

Murphy, P. (2010). Web-based collaborative reading exercises for learners in remote locations: the effects of computer-mediated feedback and interaction via computer-mediated communication. *ReCALL, 22*(2), 112–134. https://doi.org/10.1017/S0958344010000030

Murray, J., & Epstein, L. H. (1981). Improving oral hygiene with videotape modeling. *Behavior Modification, 5*, 360–371. https://doi.org/10.1177/014544558153005

Nagata, N. (1993). Intelligent computer feedback for second language instruction. *Modern Language Journal,* 77, 330–339. https://doi.org/10.1111/j.1540–4781.1993.tb01980.x

Nagata, N., & Swisher, M. V. (1995). A Study of Consciousness-Raising by Computer: *The Effect of Metalinguistic Feedback on Second Language Learning. Foreign Language Annals, 28*(3), 337–347. https://doi.org/10.1111/j.1944–9720.1995.tb00803.x

Narciss, S., & Huth, K. (2006). Fostering achievement and motivation with bug-related tutoring feedback in a computer-based training for written subtraction. *Learning and Instruction, 16,* 310–322. https://doi.org/10.1016/j.learninstruc.2006.07.003

Neenan, D. M., & Routh, D. K. (1986). Response cost, reinforcement, and children's Perteus Maze qualitative performance. *Journal of Abnormal Child Psychology, 14*, 469–480. https://doi.org/10.1007/BF00915439

Neri, A., Cucchiarini, C., & Strik, H. (2008). The effectiveness of computer-based speech corrective feedback for improving segmental quality in L2 *Dutch. ReCALL, 20*, 225–243. https://doi.org/10.1017/S0958344008000724

Newman, M. I., Williams, R. G., & Hiller, J. H. (1974). Delay of information feedback in an applied setting: Effects on initially learned and unlearned items. *Journal of Experimental Education, 42,* 55–59. https://doi.org/10.1080/00220973.1974.11011494

O'Relly, L. (1999). The effect of focused versus unfocused communication tasks on the development of linguistic competence during negotiated interaction. Unpublished doctoral dissertation. University of South Florida, Tampa.

O'Relly, L. V., Flaitz, J., & Kromrey, J. D. (2001). Two modes of correcting communicative tasks: Recent findings. *Foreign Language Annals, 14*, 246–257. https://doi.org/10.1111/j.1944–9720.2001.tb02406.x

Olson, G. H. (1971). A multivariate examination of the effects of behavioral objectives, knowledge of results and the assignment of grades on the facilitation of classroom learning. Dissertation Abstracts International, 32, 6214A. (University Microfilms No. 72–13,552)

Olson, M. W., & Raffeld, P. (1987). The effects of written comments on the quality of student compositions and the learning of content. Reading Psychology: *An International Quarterly, 8*(4), 273–293. https://doi.org/10.1080/0270271870080404

Olson, V. L. B. (1990). The revising processes of sixth-grade writers with and without peer feedback. *Journal of Educational Research, 84*, 22–29. https://doi.org/10.1080/00220671.1990.10885987

Ozolins, D. A., & Anderson, R. P. (1980). Effects of feedback on the vigilance task performance of hyperactive and hypoactive children. *Perceptual and Motor Skills, 50*, 415–424. https://doi.org/10.2466/pms.1980.50.2.415

Paige, D. D. (1966). Learning while testing. *Journal of Educational Research, 59*, 276–277. https://doi.org/10.1080/00220671.1966.10883355

Pambookian, H. S. (1972). The effect of feedback from students to college instructors on their teaching behavior. *Dissertation Abstracts International, 33*. 4950A. (University Microfilms No. 73–6893)

Payne, D. A., & Hobbs, A. M. (1979). The effect of college course evaluation feedback on instructor and student perceptions of instructional climate. *Higher Education, 8*, 525–533. https://doi.org/10.1007/BF00139792

Pearson, C. A. L. (1991). An assessment of extrinsic feedback on participation, role perceptions, motivation, and job satisfaction in a self-managed system for monitoring group achievement. *Human Relations, 4*, 517–537. https://doi.org/10.1177/001872679104400506

Peeck, J., & Tillema, H. H. (1979). Learning from feedback: Comparison of two feedback procedures in a classroom setting. *Perceptual and Motor Skills, 48,* 351–354. https://doi.org/10.2466/pms.1979.48.2.351

Peeck, J., Van Den Bosch, A. B., & Kreupeling, W. J. (1985). Effects of informative feedback in relation to retention of initial responses. *Contemporary Educational Psychology, 10(*4), 303–313. https://doi.org/10.1016/0361–476X(85)90028–1

Peña, E., Quinn, R., & Iglesias, A. (1992). The application of dynamic methods to language assessment: A non-biased procedure. *The Journal of Special Education, 26*, 269–280. https://doi.org/10.1177/002246699202600304

Perkins, V. L. (1988). Feedback effects on oral reading errors of children with learning disabilities. *Journal of Learning Disabilities, 21,* 244–248. https://doi.org/10.1177/002221948802100412

Philippakos, Z. (2012). Effects of reviewing on fourth- and fifth-grade students' persuasive writing and revising (Unpublished doctoral dissertation). University of Delaware, Newark.

Phillips, J. S., & Freedman, S. M. (1985). Contingent pay and intrinsic task interest: Moderating effects of work values. *Journal of Applied Psychology, 70*(2), 306–313. https://doi.org/10.1037/0021–9010.70.2.306

Phillips, J. S., & Lord, R. G. (1980). Determinants of intrinsic motivation: Locus of control and competence information as components of Deci's cognitive evaluation theory. *Journal of Applied Psychology,* 65(2), 211. https://doi.org/10.1037/0021–9010.65.2.211

Phye, G., & Baller, W. (1970). Verbal retention as a function of the informativeness and delay of the informative feedback: A replication. *APA Experimental Publication System, 7,* MS-232–4. https://doi.org/10.1037/h0029798

Pinder, G. C. (1976). Additivity versus non-additivity of intrinsic and extrinsic incentives: Implications for work motivation, performance, and attitudes. *Journal of Applied Psychology, 61*, 693–700. https://doi.org/10.1037/0021–9010.61.6.693

Pittman, T. S., Cooper, E. E., & Smith, T. W. (1977). Attribution of causality and the overjustification effect. *Personality and Social Psychology Bulletin, 3,* 280–283. https://doi.org/10.1177/014616727700300226

Pittman, T. S., Davey, M. E., Alafat, K. A., Wetherill, K. V., & Kramer, N. A. (1980). Informational versus controlling verbal rewards. *Personality and Social Psychology Bulletin, 6*(2), 228–233. https://doi.org/10.1177/014616728062007

Pittman, T. S., Emery, J., & Boggiano, A. K. (1982). Intrinsic and extrinsic motivational orientations: Reward-induced changes in preference for complexity. *Journal of Personality and Social Psychology, 42,* 789–797. https://doi.org/10.1037/0022–3514.42.5.789

Polahar, K. L. (1982). The effect of student feedback to college business instructors on the evaluation of instruction. *Dissertation Abstracts International, 43*, 2208A. (University Microfilms No. 82- dt535)

Polio, C., Fleck, C., & Leder, N. (1998). "If I only had more time:" ESL learners' changes in linguistic accuracy on essay revisions. *Journal of Second Language Writing, 7*, 43–68. https://doi.org/10.1016/S1060–3743(98)90005–4

Porac, J. F., & Meindl, J. (1982). Undermining overjustification: Inducing intrinsic and extrinsic task representations. *Organizational Behavior and Human Performance, 29*, 208–226. https://doi.org/10.1016/0030-5073(82)90256–2

Prater, D. L., & Bermudez, A. B. (1993). Using peer response groups with limited English proficient writers. *Bilingual Research Journal, 17,* 99 -116. https://doi.org/10.1080/15235882.1993.10162650

Prestwood, J. S., & Weiss, D. J. (1978). The effects of knowledge results and test difficulty on ability test performance and psychological reactions to testing (Research Rep. No. 78–2). Minneapolis: University of Minnesota, Department of Psychology, Psychometric Methods Program.

Pretty, G. H., & Seligman, C. (1984). Affect and the overjustification effect. *Journal of Personality and Social Psychology, 46,* 1241–1253. https://doi.org/10.1037/0022–3514.46.6.1241

Pridemore, D. R., & Klein, J. D. (1991). Control of feedback in computer-assisted instruction. *Educational Technology Research and Development, 39*, 27–32. https://doi.org/10.1007/BF02296569

Pridemore, D. R., & Klein, J. D. (1995). Control of Practice and Level of Feedback in Computer-Based Instruction. *Contemporary Educational Psychology, 20*(4), 444–450. https://doi.org/10.1006/ceps.1995.1030

Pritchard, R, D., Campbell, K. M., & Campbell, D. (1977), Effects of extrinsicfinancialrewards on intrinsic motivation. *Journal of Applied Psychology, 62*, 9–15, https://doi.org/10.1037//0021–9010.62.1.9

Pritchard, R. D., Jones, S. D., Roth, P. L., Stuebing, K. K., & Ekeberg, S. E. (1988). Effects of group feedback, goal setting, and incentives on organizational productivity. *Journal of Applied Psychology, 73,* 337–358. https://doi.org/10.1037/0021–9010.73.2.337

Ramsey, R. S. (1981). The effect of evaluative feedback from students on faculty performance at the University of Mississippi. Dissertation Abstracts International, 47, 4622A. (University Miemfilois No. 81-08768)

Rees, P. J. (1986). Do medical students learn from multiple choice examinations?. *Medical Education, 20*(2), 123–125. https://doi.org/10.1111/j.1365–2923.1986.tb01058.x

Reid, L., Lefebvre-Pinard, M., & Pinard, A. (1988). Generalization of training speaking skills: The role of overt activity, feedback, and child's initial level of competence. *Perceptual and Motor Skills, 66*(3), 963–978. https://doi.org/10.2466/pms.1988.66.3.963

Reiss, S., & Sushinsky, L. W. (1975). Overjustification, competing responses, and the acquisition of intrinsic interest. *Journal of Personality and Social Psychology, 31*, 1116–1125. https://doi.org/10.1037/h0076936

Revesz, A. J. (2007). Focus on form in task-based language teaching: Recasts, task complexity, and L2 learning (Doctoral dissertation, Columbia University).

Ringel, B. A., & Springer, C. J. (1980). On knowing how well one is remembering: The persistence of strategy use during transfer. *Journal of Experimental Child Psychology, 29*, 322–333. https://doi.org/10.1016/0022-0965(80)90023-5

Ripple, R. E. (1963). Comparison of the effectiveness of a programmed text with three other methods of presentation. *Psychological Reports, 12,* 227–237. https://doi.org/10.2466/pr0.1963.12.1.227

Robinson, S. L., DePascale, C., & Roberts, F. C. (1989). Computer-delivered feedback in group-based instruction: Effects for learning disabled students in mathematics. *Learning Disabilities Focus, 16.* 1-14.

Rocklin, T., & Thompson, J. M. (1985). Interactive effects of test anxiety and test using cognitive evaluation theory. *Journal of Personality and Social Psychology, 45*, 736–750.

Roig-Torres, T. (1992). Error correction in the natural approach classroom: A contrastive study. Unpublished doctoral dissertation. University of Pittsburgh, Pennsylvania.

Roos, L. L., Wise, S. L., & Plake, B. S. (1997). The role of item feedback in self-adapted testing. *Educational and Psychological Measurement, 57*, 85–98. https://doi.org/10.1177/0013164497057001005

Roper, W. J. (1977). Feedback in computer assisted instruction. *Programmed Learning and Educational Technology, 4*, 44–49. https://doi.org/10.1080/1355800770140107

Rosa, E.M. & Lewo, R.P. (2004) Computerized task-based exposure, explicitness, type of feedback, and Spanish L2 development. *Modern Language Journal, 88*, 192–216. https://doi.org/10.1111/j.0026–7902.2004.00225.x

Rosenbaum, M., & Ben-Ari, K. (1985). Learned helplessness and learned resourcefulness: Effects of noncontingent success and failure on individuals differing in self-control skills. *Journal of Personality and Social Psychology, 48,* 198–215. https://doi.org/10.1037/0022–3514.48.1.198

Rosenfield, D., Folger, R., & Adelman, H. (1980). When rewards reflect competence: A qualification of the overjustification effect. *Journal of Personality and Social Psychology, 39*, 368–376. https://doi.org/10.1037/0022–3514.39.3.368

Rosenthal, B. D. (2006). Improving elementary-age children's writing fluency: A comparison of improvement based on performance feedback frequency (Unpublished doctoral dissertation). Syracuse University, Syracuse, NY.

Ross, M. (1975). Salience of reward and intrinsic motivation. *Journal of Personality and Social Psychology, 32*, 245–254. https://doi.org/10.1037/0022–3514.32.2.245

Rotem, A. (1976). The effects of feedback from students to university instructors: An experimental study. *Dissertation Abstracts International, 16,* 5877 A. (University Microfilms No. 76–658 1)

Rothkopf, E. Z. (1966). Learning from written instructive materials: An exploration of the control of inspection behavior by test-like events. *American Educational Research Journal, 3,* 241–249. https://doi.org/10.3102/00028312003004241

Russell, J. C., Studstill, O. L., & Grant, R. M. (1981). Effect of expectancies on intrinsic motivation. *Psychological Reports, 49(*2), 423–428. https://doi.org/10.2466/pr0.1981.49.2.423

Rust, J. O., Strang, H. R., & Bridgeman, B. (1977). How knowledge of results and goal setting function during academic tests. *The Journal of Experimental Education, 45*(4), 52–55. https://doi.org/10.1080/00220973.1977.11011599

Ryan, R. M. (1982). Control and information in the intrapersonal sphere: An extension of cognitive evaluation theory. *Journal of Personality and Social Psychology, 43*, 450–461. https://doi.org/10.1037/0022–3514.43.3.450

Ryan, R. M., Mims, V., & Koestner, R. (1983). Relation of reward contingency and interpersonal context to intrinsic motivation: A review and test using cognitive evaluation theory. *Journal of Personality and Social Psychology*, 45, 736–750 https://doi.org/10.1037/0022–3514.45.4.736

Ryan, R. M., Mims, V., & Koestner, R. (1983). Relation of reward contingency and interpersonal context to intrinsic motivation: A review and test using cognitive evaluation theory. *Journal of Personality and Social Psychology,* 45, 736–751 https://doi.org/10.1037/0022–3514.45.4.736

Sagarra, N. (2007). From CALL to face-to-face interaction: The effect of computer-delivered recasts and working memory on L2 development. In A. Mackey (Ed.), Conversational interaction in second language acquisition (pp. 229–248). New York: Oxford University Press.

Sanz, C., & Morgan-Short, K. (2004). Positive evidence versus explicit rule presentation and explicit negative feedback: A computer-assisted study. *Language Learning, 54,* 35–78. https://doi.org/10.1111/j.1467–9922.2004.00248.x

Sassenrath, J. M., & Gaverick, C. M. (1965). Effects of differential feedback from examinations on retention and transfer. *Journal of Educational Psychology, 56*, 259–263. https://doi.org/10.1037/h0022474

Sassenrath, J. M., & Yonge, G. D. (1968). Delayed information feedback, feedback cues, retention set, and delayed retention. *Journal of Educational Psychology*, 59, 69–73. https://doi.org/10.1037/h0025512

Sassenrath, J. M., & Yonge, G. D. (1969). Effects of delayed information feedback and feedback cues in learning and retention. *Journal of Educational Psychology, 60*, 174–177. https://doi.org/10.1037/h0027618

Sauro, S. (2007). A comparative study of recasts and metalinguistic feedback through computer mediated communication on the development of L2 knowledge and production accuracy. Unpublished doctoral dissertation. University of Pennsylvania, Philadelphia.

Schaffer, L. C., & Hannafin, M. J. (1986). The effects of progressive interactivity on learning from interactive video. *Educational Communication and Technology Journal, 34*, 89- 96.

Schloss, P. J., Wisniewski, L. A., & Cartwright, G. P. (1988). The differential effect of learner control and feedback in college students' performance on CAI modules. Journal of Educational Computing Research, 4, 141- 150. https://doi.org/10.2190/XJYY-TX9V-DHGQ-5Q50

Schmitt, N., Coyle, B. W., & Saari, B. B. (1977). Types of task information feedback in multiple-cue probability learning. *Organizational Behavior and Human Performance, 18*, 316–328. https://doi.org/10.1016/0030-5073(77)90033–2

Schuldt, W. J., & Bonge, D. (1979). Effects of self-imposition and experimenter imposition of achievement standards on performance. *Psychological Reports, 45*, 19–122. https://doi.org/10.2466/pr0.1979.45.1.119

Schunk, D. H., & Swartz, C. W. (1993a). Goals and progress feedback: Effects on self-efficacy and writing achievement. *Contemporary Educational Psychology, 18*, 337–354. https://doi.org/10.1006/ceps.1993.1024

Scott, C. A., & Yalch, R. F. (1978). A test of the self-perception explanation of the effects of rewards on intrinsic interest. *Journal of Experimental Social Psychology, 14*(2), 180–192. https://doi.org/10.1016/0022–1031(78)90024-0

Scott, N. H. (1976). A pilot study testing the effects of feedback on teaching behavior as measured by student ratings at New River Community College, Dublin, Virginia. *Dissertation Abstracts International, 37,* 1950A. (University Microfilms No. 76–23235)

Scott, W. ., Farh, J.-L., & Podsakoff, P. M. (1988). The effects of "intrinsic" and "extrinsic" reinforcement contingencies on task behavior. *Organizational Behavior and Human Decision Processes, 41*(3), 405–425. https://doi.org/10.1016/0749–5978(88)90037–4

Semke, H. M. (1980). The comparative effects of four methods of treating free-writing assignments on the second language skills and attitudes of students in college level first year German (Unpublished doctoral dissertation). University of Minnesota, Minneapolis, MN.

Sengupta, S. (2000). An investigation into the effects of revision strategy instruction on L2 secondary school learners. *System, 28*(1), 97–113. https://doi.org/10.1016/S0346–251X(99)00063–9

Sepe, T. D. (1974). An experimental study of the impact of instructor evaluation on classroom teaching performance.

Shavit, H., & Rabinowitz, A. (1978). Locus of control and effects of failure on performance and perceived competence. *Journal of Personality Assessment, 42*, 265–271. https://doi.org/10.1207/s15327752jpa4203_7

Sheen, Y. (2007). The effect of focused written corrective feedback and language aptitude on ESL learners' acquisition of articles. *TESOL Quarterly, 41,* 255–283. https://doi.org/10.1002/j.1545–7249.2007.tb00059.x

Sheen, Y., Wright, D., & Moldawa, A. (2009). Differential effects of focused and unfocused written correction on the accurate use of grammatical forms by adult ESL learners. *System, 37,* 556–569. https://doi.org/10.1016/j.system.2009.09.002

Shintani, N., & Ellis, R. (2013). The comparative effect of direct written corrective feedback and meta-linguistic explanation on learners' explicit and implicit knowledge of the English indefinite article. *Journal of Second Language Writing, 22,* 286–306. https://doi.org/10.1016/j.jslw.2013.03.011

Simensen, R. J. (1973). Acquisition and retention of a motor skill by normal and retarded students. *Perceptual and Motor Skills, 36,* 791- 799. https://doi.org/10.2466/pms.1973.36.3.791

Simmons, R. W, & Snyder, R. J. (1982). The effects of filling a variable knowledge of results-delay interval upon the acquisition and retention of a ballistic skill. Unpublished manuscript, San Diego State University.

Skeff, K. M. (1983). Evaluation of a method for improving the teaching performance of attending physicians. *American Journal of Medicine, 75*. 465–470. https://doi.org/10.1016/0002–9343(83)90351-0

Smith, D. L. (1977). The relationship of feedback to professors of the results of student mtings of their teaching effectiveness at mid- semester to their end-of-semester ratings. (Doctoral dissertation, University of Southern California). Dissertation Abstracts International, 38. 187A.

Sobal, J., & Juhasz, J. B. (1977). Sex, experimenter, and reinforcement effects in verbal learning. *Journal of Social Psychology, 102,* 267–273. https://doi.org/10.1080/00224545.1977.9713273

Sonnenschein, S. (1986). Developing referential communication: Transfer across novel tasks. *Bulletin of the Psychonomics Society,* 24, 127–130. https://doi.org/10.3758/BF03330524

Steele, A L. (1971). Contingent socio-music listening periods in a preschool setting. *Journal of Music Therapy, 8,* 131–139. https://doi.org/10.1093/jmt/8.4.131

Stinson, M., & Belmont, J. M. (1979). Effects of feedback manipulation and feedback preference upon children's performance. *Journal of Genetic Psychology, 155*, 159–160. https://doi.org/10.1080/00221325.1979.10533429

Strang, H. R. (1983). The effects of knowledge of results upon reaction time performance. *Journal of General* Psychology, 108, 11–17. https://doi.org/10.1080/00221309.1983.9711473

Strang, H. R., & Rust, J. O. (1973). The effects of immediate knowledge of results and task definition on multiple-choice answering. *The Journal of Experimental Education, 42*(1), 77–80. https://doi.org/10.1080/00220973.1973.11011449

Strang, H. R., Lawrence, E. C., & Fowler, P. C. (1978). Effects of assigned goal level and knowledge of results on arithmetic computation: A laboratory study. *Journal of Applied Psychology, 63*, 446–450. https://doi.org/10.1037/0021–9010.63.4.446

Sturges, P. T. (1969). Verbal retention as a function of the informativeness and delay of informative feedback. *Journal of Educational Psychology, 60*, 11–14. https://doi.org/10.1037/h0026638

Sturges, P. T. (1972). Information delay and retention: Effect of information in feedback and tests. *Journal of Educational Psychology, 63,* 32–43. https://doi.org/10.1037/h0032158

Sturges, P. T. (1978). Delay of informative feedback in computer-assisted testing. *Journal of Educational Psychology, 70*, 378–387. https://doi.org/10.1037/0022-0663.70.3.378

Sullivan, H. J., Schutz, R. E., & Baker, R. L. (1971). Effects of systematic variations in reinforcement contingencies on learner performance. *American Educational Research Journal, 8*, 135–142. https://doi.org/10.3102/00028312008001135

Sun, S. (2013). Written corrective feedback: Effects of focused and unfocused grammar correction on the case acquisition in L2 German (Unpublished doctoral dissertation). University of Kansas, Lawrence, KS.

Surber, J. R., & Anderson, R. C. (1975). Delay-retention effect in natural classroom settings. *Journal of Educational Psychology, 67,* 170–173. https://doi.org/10.1037/h0077003

Swann, W. B., & Pittman, T. S. (1977). Initiating play activity of children: The moderating influence of verbal cues on intrinsic motivation. *Child Development,* 48, 1128–1132. https://doi.org/10.2307/1128374

Tait, K., Hartley, J. R., & Anderson, R. C. (1973). Feedback procedures in computer-assisted arithmetic instruction. *British Journal of Educational Psychology, 43*, 161–171. https://doi.org/10.1111/j.2044–8279.1973.tb00752.x

Takashima, H. (1995). A study of focused feedback, or output enhancement, in promoting accuracy in communicative activities. Unpublished doctoral dissertation. Temple University, Philadelphia, PA.

Tennyson, R. D. (1980). Instructional control strategies and content structure as design variables in concept acquisition using computer-based instruction. *Journal of Educational Psychology, 72*(4), 525–532. https://doi.org/10.1037/0022-0663.72.4.525

Tennyson, R. D. (1981). Use of adaptive information for advisement in learning concepts and rules using computer-assisted instruction. *American Educational Research Journal, 18,* 425–438. https://doi.org/10.3102/00028312018004425

Tennyson, R. D., & Buttrey, T. (1980). Advisement and management strategies as design variables in computer-assisted instruction. *ECTJ, 28(*3), 169.

Tindale, R. S., Kulik, C. T., & Scott, L. A. (1991). Individual and group feedback and performance: An attributional perspective. *Basic and Applied Social Psychology, 12*, 41–62. https://doi.org/10.1207/s15324834basp1201_4

Titus, T. G. (1973). Continuous feedback in recognition memory. *Perceptual and Motor Skills, 37*, 771–776. https://doi.org/10.1177/003151257303700320

Tobias, S. (1984). Macroprocesses, individual differences, and instructional methods. Paper presented at the annual meeting of the American Educational Research Association, New Orleans. (ERIC Document Reproduction Service No. ED 259 019)

Tomasello, M., & Herron, C. (1989). Feedback for language transfer errors: The garden path technique. *Studies in Second Language Acquisition, 11*, 385–395. https://doi.org/10.1017/S0272263100008408

Truscott, J., & Hsu, A. Y. (2008). Error correction, revision, and learning. *Journal of Second Language Writing, 17*, 292–305. https://doi.org/10.1016/j.jslw.2008.05.003

Tsao, B. L. (1977). The effects of error rate, knowledge of correct results, and test anxiety in learning. *Dissertation Abstarcts International,, 29*, 1426A (University Microfilms No. 78–15, 385)

Turner, R. R. (1975). Effect of information feedback on matrices learning for differing socioeconomic levels. *Journal of Educational Psychology, 67*, 285–295. https://doi.org/10.1037/h0076931

Tzuriel, D., & Caspi, N. (1992). Cognitive modifiability and cognitive performance of deaf and hearing preschool children. *The Journal of Special Education, 26,* 235–252. https://doi.org/10.1177/002246699202600302

Valdez, A. J. (2009). Encouraging mindful feedback processing: Computer-based instruction in descriptive statistics (Doctoral dissertation). Available from ProQuest Dissertations and Theses database. (UMI No. 3329482)

Van Beuningen, C. G., de Jong, N. H., & Kuiken, F. (2008). The effect of direct and indirect corrective feedback on L2 learners' written accuracy. *ITL International Journal of Applied Linguistics, 156*, 279- 296. https://doi.org/10.2143/ITL.156.0.2034439

Van Dyke, B. F., & Newton, J. M. (1972). Computer-assisted instruction: Performance and attitudes. *Journal of Educational Research, 65*, 291–293. https://doi.org/10.1080/00220671.1972.10884321

Vellella, J. A. (1996). The effectiveness of curriculum-based measurement on spelling achievement: A comparison of two procedures (Unpublished master's thesis). Illinois State University, Normal.

Vispoel, W. P. (1998). Psychometric characteristics of computer-adaptive and self-adaptive vocabulary tests: The role of answer feedback and test anxiety. *Journal of Educational Measurement, 35*, 155–167. https://doi.org/10.1111/j.1745–3984.1998.tb00532.x

Vitulli, W. F. (1982). Effects of immediate feedback on computer-assisted testing of ESP performance. *Psychological Reports, 52,* 403- 408. https://doi.org/10.2466/pr0.1982.51.2.403

Wade-Stein, D., & Kintsch, E. (2004). Summary Street: Interactive computer support for writing. *Cognition and Instruction, 22*(3), 333–362. https://doi.org/10.1207/s1532690xci2203_3

Wade, M. G., & Newell, K. M. (1972). Performance Criteria for Stabilometer Learning. *Journal of Motor Behavior, 4*(4), 231–239. https://doi.org/10.1080/00222895.1972.10734939

Wade, T. C. (1974). Relative effects on performance and motivation of self-monitoring correct and incorrect responses. *Journal of Experimental Psychology, 103*(2), 245–248. doi:10.1037/h0036847 https://doi.org/10.1037/h0036847

Wagner, S.U. (1983). The effect of immediacy and type of informative feedback on retention in a computer-assisted task (unpublished doctroal dissertation). The Florida State University, Tallahassee.

Weerts, R. R. (1978). The use of feedback from student ratings for improving college leaching. (Research Report No. 6, Student Perceptions of Teaching [SPOT]: VIII). Iowa City: University of Iowa, Evaluations Examination Service.

Weiner, M. J. (1980). The effect of incentive and control over outcomes upon intrinsic motivation and performance. *Journal of Social Psychology, 112*, 247–254. https://doi.org/10.1080/00224545.1980.9924326

Welsh, P., Antoinetti, J. A., & Thayer, P. W. (1965). An industrywide study of programmed instruction. *Journal of Applied Psychology,* 49, 61–73. https://doi.org/10.1037/h0021694

Wiener, E. L. (1974). An adaptive vigilance task with knowledge of results. *Human Factors, 26*, 333–338. https://doi.org/10.1177/001872087401600401

Wiener, E. L. (1975). On simultaneous monitoring and tracking. *Applied Psychology, 60*, 100–105. https://doi.org/10.1037/h0076366

Wiener, E. L., & Attwood, D. A. (1968). Training for vigilance: Combined cueing and knowledge of results. *Journal of Applied Psychology, 52*(61), 474–478. https://doi.org/10.1037/h0026444

Williams, B. W. (1980). Reinforcement, behavior constraint, and the overjustification effect. *Journal of Personality and Social Psychology, 39,* 599–614. https://doi.org/10.1037/0022–3514.39.4.599

Wimperis, B. & Farr, J, (1979), The effects of task content and reward contingency upon task performance and satisfaction. *Journal of Applied Social Psychology, 9,* 229–249. https://doi.org/10.1111/j.1559–1816.1979.tb02708.x

Winett, R. A., & Vachon, E. M. (1974). Group feedback and group contingencies in modifying behavior of fifth graders. *Psychological Reports, 34*, 1283–1292.

Wise, S. L., Plake, B. S., Pozehl, B. J., Barnes, L. B., & Lukin, L. E. (1989). Providing item feedback in computer-based tests: Effects of initial success and failure. *Educational and Psychological Measurement, 49,* 479–486. https://doi.org/10.1177/0013164489492021

Wise, S. L., Plake, B. S., Pozehl, B. J., Barnes, L. B., & Lukin, L. E. (1989). Providing item feedback in computer-based tests: Effects of initial success and failure. *Educational and Psychological Measurement, 49*(2), 479–486. https://doi.org/10.1177/0013164489492021

Wise, W. (1992). The effects of revision instruction on eighth graders' persuasive writing (Unpublished doctoral dissertation). University of Maryland, College Park.

Wolter, D. R. (1975). Effect of feedback on performance on a creative writing task (Unpublished doctoral dissertation). University of Michigan, Ann Arbor.

Xiang, W. (2004). Encouraging self-monitoring in writing by Chinese students. *ELT Journal, 58*, 238–246. https://doi.org/10.1093/elt/58.3.238

Xu, M. (2009). An investigation of the effectiveness of intelligent elaborative feedback afforded by pedagogical agents on improving young Chinese language learners' vocabulary acquisition (doctoral dissertation). Available from ProQuest Dissertations and Theses database. (UMI No. 3359045)

Zimmerman, B. J., & Kitsantas, A. (2002). Acquiring writing revision and self-regulatory skill through observation and emulation. *Journal of Educational Psychology, 94,* 660–668. https://doi.org/10.1037/0022-0663.94.4.660

Zohar, D., Cohen, A., & Azar, N. (1980). Promoting increased use of ear protectors in noise through information feedback. *Human Factors, 22,* 69–79. https://doi.org/10.1177/001872088002200108
